# Cooperative decision-making in borderline personality disorder: insights from a preregistered study using a comprehensive economic task battery

**DOI:** 10.1186/s40479-025-00295-2

**Published:** 2025-06-17

**Authors:** L. M. Doppelhofer, J. Löloff, C. Neukel, S. C. Herpertz, C. W. Korn

**Affiliations:** 1 Department of General Psychiatry, Center for Psychosocial Medicine, Heidelberg University, Heidelberg, Germany; 2 German Center for Mental Health (DZPG), partner site Mannheim/Heidelberg/Ulm, Germany

**Keywords:** Borderline Personality Disorder, Economic Games, Cooperation, Fairness

## Abstract

**Background:**

Social decision-making poses challenges for individuals with Borderline Personality Disorder (BPD), which can lead to maladaptive interpersonal functioning. Although previous research on cooperative decisions in BPD has produced mixed findings, studies examining different aspects of cooperative behavior suggest that individuals with BPD may specifically show reduced reactive cooperation, while their active cooperation appears similar to that of healthy individuals.

**Methods:**

To address these mixed results, we used a comprehensive battery of economic tasks in this preregistered study to assess different aspects of cooperation in 35 women with BPD and 50 healthy women.

**Results:**

Consistent with our expectations, there were no significant differences between groups in active cooperation within the Dictator Game. However, contrary to our hypotheses, neither BPD status nor dissociality scores were related to reactive cooperation in the Ultimatum Game. To synthesize findings across studies, a mini meta-analysis was conducted, revealing no significant overall effect of group membership on rejection rates in the Ultimatum Game. Additionally, women with BPD showed similar social preferences, fairness perceptions and inequality aversion as the control group.

**Conclusion:**

These results suggest that in the context of anonymous, one-shot economic games, individuals with BPD may not differ from healthy individuals in terms of cooperative behavior. This highlights the importance of using baseline variants of standard economic tasks when considering contextual factors that affect social behavior in BPD.

**Supplementary Information:**

The online version contains supplementary material available at 10.1186/s40479-025-00295-2.

## Introduction

Individuals often face situations where their decisions have ripple effects on others. The ability to make appropriate social decisions and cooperate is essential for successful social interactions and relationships. Mental disorders like Borderline Personality Disorder (BPD) can alter how individuals perceive social cues and react in social situations. BPD is characterized by symptoms including intense negative affect, impulsive behavior, unstable relationships, and an unstable self-image [1]. These features are linked to deficits in various processes that are critical for social functioning.

Individuals diagnosed with BPD hold a more inconsistent and negative view of themselves [2–4] and extend this negativity to the social world around them, which they perceive as more negative and malevolent compared to those without the disorder [5, 6]. They also tend to evaluate other people as more negative [3], aggressive [7], less trustworthy [8, 9], and anticipate greater selfishness in others’ behavior [10] compared to healthy individuals. Relatedly, individuals with BPD have been shown to exhibit higher rejection sensitivity [11–14] and feel reduced connectedness with others even when being included [15–18]. These challenges impact both the quantity and quality of social relationships. Individuals with BPD tend to maintain fewer social connections, and their relationships are often characterized by greater instability and perceived ambivalence and negativity compared to those of healthy and clinical controls [19–21].

To investigate complex social behavior, economic games provide a useful tool, offering controlled settings to assess cooperation and decision-making. While different economic games have been widely used in BPD research (for reviews see [22, 23]), the findings remain inconsistent.

For example, findings from economic game studies suggest that individuals with BPD struggle to sustain and repair broken cooperation compared to controls, particularly in Trust Game (TG) paradigms [24–26]. Similar patterns have been observed in other paradigms, such as the Prisoner’s Dilemma [27] and the Ultimatum Game (UG) among individuals with elevated BPD features [28]. However, these patterns are not consistently replicated: while some studies report no overall group differences in prosocial preferences [10, 28, 29], others note inconsistent trust and punishment behavior in individuals with BPD [30, 31], or point to context-specific impairments, particularly following positive social interactions [17, 32, 33].

A review by Jeung et al. [22] suggested that individuals with BPD tend to act in a more self-interested and economically rational manner in these paradigms. But overall mixed findings suggest the need for a more nuanced framework. One promising direction is the distinction between *reactive cooperation* (e.g., non-retaliation and the ability to forgive after unfair treatment) and *active cooperation* (e.g., non-exploitation and a willingness to share or give) [28, 34, 35]. Research indicates that borderline personality features are associated with deficits in reactive cooperation but not necessarily in active cooperation [28, 34]. This may explain why individuals with BPD typically perform similarly to controls in tasks like the Social Value Orientation (SVO) task or the Dictator Game (DG) but diverge in contexts like the UG or the trustee role in the TG, which require reactive responses.

To better understand the mechanisms underlying reduced cooperation in their TG, King-Casas et al. [25] proposed that individuals with BPD may struggle to recognize or interpret norm violations [17, 25]. Unfair offers may confirm negative expectations, making it less likely that such behaviors are perceived as violations [10, 17, 25]. These results are complemented by studies reporting that individuals with BPD also fail to respond cooperatively after positive interactions [17, 32]. A related account suggests that these impairments stem from a failure to recognize fair or positive interactions [32], which may, in turn, be influenced by exaggerated ideas about fairness and justice [36]. Consistent with this view, Lis et al. [37] found that BPD traits are associated with heightened justice sensitivity—directed both toward oneself and toward others. Justice sensitivity is specifically linked to perceiving others’ behavior as unfair [38], and may thus influence fairness perception in economic games. Fairness perception, in turn, is closely related to inequality aversion—a preference against unequal outcomes [39]. We therefore propose that individuals with BPD may also exhibit heightened inequality aversion. However, this hypothesis remains to be systematically tested, as inequality aversion has not yet been directly studied in the context of BPD. Notably, some studies report no differences between individuals with and without BPD in fairness perception or recognition of norm violations [30, 40–42], highlighting the need for further research to clarify these inconsistencies.

In sum, prior research highlights that cooperative behavior in individuals with BPD varies considerably across social exchange paradigms, depending on the specific task structure, the individual’s role within the interaction, and the situational context. Adding complexity, specific personality traits may modulate responses to different situational affordances [43, 44]. In particular, Hepp and Niedtfeld [44] propose that the ICD-11 personality trait dissociality—marked by reduced empathy, increased hostility, and a tendency to exploit others [45]–may contribute to reduced prosocial behavior in situations that entail negative reciprocity.

### Hypotheses

**Fig. 1:**
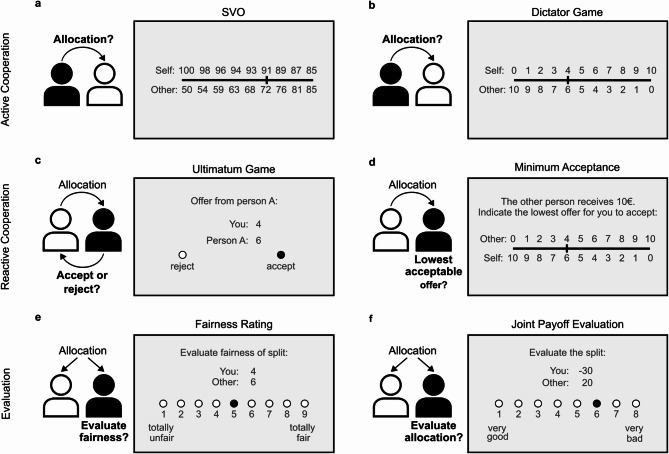
Overview of the tasks and concepts. The first row depicts active cooperation tasks, the second row depicts reactive cooperation tasks, and the third row depicts evaluations of hypothetical allocations to measure fairness perception and inequality aversion. The black icon represents the participant and the white icon the anonymous partner. The arrows indicate how the resources are being allocated. The bold text near the icons signifies the participant’s specific task assignment in the depicted scenario. **a** Example item of the Social Value Orientation (SVO) task to measure general social value orientation and a categorical classification (altruistic, prosocial, individualistic, or competitive). Participants were asked to choose one of nine predefined options to allocate money between themselves and another participant. **b** In the Dictator Game (DG) participants were in the role of the proposer and had to allocate 10€ between themselves and another participant. **c** Example offer in the Ultimatum Game (UG). Participants acting as responders, had to decide whether to accept or reject offers from six anonymous participants. Accepting an offer resulted in both participants receiving the proposed allocation, whereas rejecting it meant neither party received any payout. **d** In the Minimum Acceptance Rating question, participants were asked to indicate the minimum amount they would require the other participant to offer for them to accept the split of 10€. **e** Example item of the Fairness Ratings. Participants had to assess the fairness of 11 hypothetical offers using the same allocation options as in the DG and UG. **f** Example item of the Joint Payoff Evaluation (JPE) task. In this task, participants rated payoff pairs (ranging from − 50 to 50) for themselves and another participant

To address the mixed findings of previous studies on cooperative behavior in individuals with BPD, we used a wide set of economic games with a focus on simple one-shot tasks (Fig. 1) to test a well characterized sample of BPD patients and a healthy control (HC) group on their social preferences, their ability to cooperate in active and reactive situations, as well as their fairness perception and inequality aversion. Based on previous literature, we expected no differences in active cooperation but impairments in reactive cooperation and an altered inequality aversion and fairness perception in individuals with BPD compared to a control group. We tested active cooperation using two different tasks, in which participants actively allocate money between themselves and another anonymous person: the Social Value Orientation (SVO) Slider Measurement [46] and the Dictator Game (DG; [47, 48]). To assess reactive cooperation, participants were engaged in an Ultimatum Game (UG; [49]) in the role of the recipient and had to decide whether to accept or reject an offer from an anonymous person. Taking a dimensional approach, we investigated whether the maladaptive trait of dissociality as assessed by the German version of the Personality Inventory for ICD-11 (PiCD [45, 50]) is associated with reduced reactive cooperation (reciprocal fairness) as suggested by Hepp and Niedtfeld [44]. To examine the reliability of choices in the UG, participants were asked to indicate the minimum amount they would accept from an anonymous proposer. To differentiate between decisions that participants actually make and decisions that participants consider fair, we explicitly prompted them to assess the fairness of various hypothetical offers using the same allocation options as in the DG and UG. In addition, we employed a computational modeling approach on an adapted version of a Joint Payoff Evaluation (JPE) task [51], wherein participants evaluated a broader range of allocation pairs. This allowed us to better understand the mechanisms governing social preferences and test potential group differences between specific parameters.

We state our preregistered (https://osf.io/jkvh8) hypotheses in the following numbered list.

- *H1*. We hypothesized no group differences in social value orientation (primary items of SVO task) because we did not expect differences in active cooperation (non-exploitation) between individuals with and without BPD.
- *H2*. We expected individuals with BPD to be more sensitive to inequality and therefore hypothesized a higher inequality aversion (and lower joint gain maximization) in the secondary items of the SVO task compared to the control group.
- *H3*. We expected that our findings would replicate previous research [28, 43] leading to the hypothesis of no group differences in a Dictator Game (active cooperation).
- *H4*. We expected reduced reactive cooperation in individuals with BPD and therefore, hypothesized a higher rate of offer rejections in the Ultimatum Game in the BPD group compared to the control group.
- *H5*. In line with our expectation of impaired reactive cooperation among individuals with BPD, we hypothesized that individuals with BPD would require a higher offer compared to controls to accept an allocation.
- *H6*. We hypothesized that the BPD group would judge fair offers as less fair than individuals in the control group would.
- *H7.1*. We postulated that the Fehr-Schmidt model [39], which offers a mathematical framework that considers own gain, disadvantageous inequality, and advantageous inequality, would provide the best fit for both groups’ data in the Joint Payoff Evaluation task.
- *H7.2*. Given our expectation of heightened inequality aversion among individuals with BPD, we hypothesized greater weights on the disadvantageous inequality aversion parameter of the Fehr-Schmidt model in the BPD group compared to the control group.
- *H8*. We hypothesized that higher dissociality scores would correlate with higher rejection rates in the Ultimatum Game (situational affordance of negative reciprocity, see [44]).

Since we could not replicate previous findings on higher rejection rates in the UG in the BPD group compared to the HC group [28, 32], we conducted a mini meta-analysis [52] to clarify the overall evidence and synthesize the results across relevant studies. The meta-analysis was not part of our preregistration plan.

## Methods

### Ethics and preregistration

This study is part of a larger project on social decision making in women with BPD. All participants also took part in another behavioral experiment and some participants took part in an fMRI experiment, which will be reported elsewhere. We preregistered this study on June 1, 2023, on the Open Science Framework (https://osf.io/jkvh8) after collection but before any analyses of the data. We analyzed the PiCD questionnaire [45, 50], which includes the dissociality scores, as part of other experiments that are preregistered separately (https://osf.io/jwky9 and https://osf.io/sd2tm). We did not observe any of the behavioral data (SVO task, JPE task, DG, UG, Minimum Acceptance Rating, Fairness Ratings) before uploading our preregistration. Changes in the protocol are reported in the Methods section.

### Participants

We recruited female participants between 18 and 40 years of age who are fluent in German. Control participants were matched for age during the recruitment process. All participants underwent a prescreening via a telephone interview and were invited to the study if they met our inclusion criteria. They were briefed on the study procedure and gave written informed consent before participating. During the in-person assessment, participants took part in a diagnostic interview, a brief cognitive task, and a set of self-report questionnaires (see Supplementary Table S1 for the full list) via the German survey system SoSci Survey [53]. All participants were debriefed at the end of the study. Refer to the Supplementary Material (section *Supplementary Methods*) for details on our power analysis, the preregistered inclusion and exclusion criteria, recruitment of participants and reimbursement.

#### Clinical assessments

We assessed Axis I disorders with the short version of the open access Diagnostic Interview for Mental Disorders (Mini-DIPS Open Access; [54]) and BPD diagnostic criteria with the German version of the International Personality Disorder Examination (IPDE; [55, 56]). The clinical interview was conducted by trained and supervised raters and typically lasted approximately 3 h. Additionally, participants underwent a three-minute speeded reasoning test (mini-q; [57]) to estimate their general cognitive abilities. Furthermore, we assessed depressive symptoms with the Beck Depression Inventory-II (BDI-II; [58]), a General Severity Index (GSI) for self-reported psychopathological symptoms experienced in the previous seven days with the Brief Symptom Checklist (BSCL; [59]), the severity of BPD with the Borderline Symptom List-23 (BSL-23; [60]), impairments of personality functioning with the Level of Personality Functioning Scale-Brief Form (LPFS-BF; [61, 62]), and personality dysfunction with the Personality Inventory for the DSM-5-Brief Form (PID-5-BF; [63, 64]).

#### Sample description

We decided to analyze all valid datasets (according to our inclusion and exclusion criteria) for the current study and included 35 patients with BPD and 50 controls in our data analysis. Age ranged from 18 to 39 in both groups and did not differ significantly between the groups (*M*_*BPD*_ = 25.8, *SD*_*BPD*_ = 5.58, *M*_*HC*_ = 24.88, *SD*_*HC*_ = 5.43, Welch’s two-sample t-test *t*_*72.035*_ = 0.756, *p* = 0.452, 95% *CI* [−1.505, 3.345], *d = −0.167*; Wilcoxon rank sum test *z* = 0.842, *p* = 0.4, *r* = 0.092). Groups did not differ in their general cognitive abilities (mini-q; M_*BPD*_ = 34.03, SD_*BPD*_ = 12.12, M_*HC*_ = 35.74, SD_*HC*_ = 9.91, Welch’s two-sample t-test *t*_*63.579*_ = −0.689, *p* = 0.493, 95% *CI* = [−6.671, 3.248], *d* = −0.155; Wilcoxon rank sum test *z* = −0.541, *p* = 0.589, *r* = 0.059). The most common school-leaving certificate among participants was a high school diploma. However, a higher percentage of participants in the HC group (98%) completed high school compared to the BPD group (74.29%). Employment status also differed between the groups: Most participants in the BPD group were currently employed (28.57% in the BPD group vs. 18% in the HC group) while the majority of participants in the HC group were students (74% in the HC group vs. 20% in the BPD group). For detailed demographic data, refer to Table 1. For the BPD group we included participants who met the threshold of five IPDE criteria and participants with sub-threshold BPD who met at least four IPDE criteria and had a prior BPD diagnosis. Within the BPD group, 32 participants had received a formal BPD diagnosis prior to participation in our study and for three participants, suspicion of a BPD diagnosis had been raised by either their psychiatrist or psychotherapist. In the BPD group 88.57% of participants fulfilled criteria of comorbid diagnoses according to the mini-DIPS interview (Table 2). About 51% of participants in the BPD group were taking some form of psychotropic medication, primarily antidepressants (Table 2). Information on current medication intake was self-reported by the participants. The BPD group and the HC group differed significantly in BDI-II, BSCL, BSL-23, IPDE, LPFS, and PID-5-BF (see Table 3 for details).

**Table 1:** Demographic characteristics of both groups

	BPD (*N* = 35)		HC (*N* = 50)	
	*N*	%	*N*	%
Education				
Secondary general school (9 years)	1	2.86	0	0
Intermediate school (10 years)	8	22.86	1	2
High school diploma (13 years)	26	74.29	49	98
Professional qualification				
Non, in professional training	9	25.71	28	56
Non, not in professional training	5	14.29	0	0
Apprenticeship	6	17.14	1	2
Vocational school	10	28.57	3	6
Technical school	0	0	1	2
University	5	14.29	17	34
Employment status				
Student	7	20	37	74
In training	6	17.14	0	0
Employed	10	28.57	9	18
Unemployed	5	14.28	2	4
Retired or homemaker	2	5.71	0	0
Other	5	14.29	2	4

**Table 2:** Comorbid diagnoses and medication intake in the BPD group

	*N*	%
Comorbid Diagnosis		
Panic disorder	4	11.43
Agoraphobia with panic	1	2.86
Specific phobia	6	17.14
Social anxiety disorder	15	42.86
Major depressive episode	2	5.71
Major depressive disorder, recurrent	17	48.57
Persistent depressive disorder	3	8.57
Obsessive-compulsive disorder	1	2.86
Body dysmorphic disorder	1	2.86
Posttraumatic stress disorder	12	34.29
Bulimia nervosa	1	2.86
Binge eating disorder	2	5.71
Illness anxiety disorder	2	5.71
Substance abuse, alcohol	1	2.86
Substance abuse, cannabis	2	5.71
No Axis I diagnosis	4	11.43
Medication		
Antidepressant, SNRI	5	14.29
Antidepressant, SSRI	10	28.57
Antidepressant, atypical	1	2.86
Atypical antipsychotics	3	8.57
Mood stabilizer (Lamotrigine)	2	5.71
No medication	17	48.57

Comorbid diagnoses were tested with the mini-DIPS diagnostic interview

**Table 3:** Clinical characteristics of both groups

Questionnaire	BPD (*N* = 35)		HC (*N* = 50)		Welch’s t-test						Wilcoxon rank sum test		
	*M*	*SD*	*M*	*SD*	*t*	*DF*	*CI (LL)*	*CI (UL)*	*p*	*d*	*z*	*p*	*r*
BDI-II	27.743	12.363	3.720	4.071	11.083	39.201	19.639	28.406	**< 0.001**	2.610	7.443	**< 0.001**	0.807
BSCL	1.584	0.737	0.186	0.261	10.764	40.003	1.136	1.661	**< 0.001**	2.530	7.426	**< 0.001**	0.805
BSL-23	1.836	0.818	0.212	0.299	11.226	40.412	1.332	1.916	**< 0.001**	2.636	7.538	**< 0.001**	0.818
IPDE	13.657	2.114	0.160	0.548	36.921	37.219	12.757	14.238	**< 0.001**	8.742	8.473	**< 0.001**	0.919
LPFS	35.629	5.309	18.840	5.068	14.618	71.135	14.499	19.078	**< 0.001**	3.235	7.523	**< 0.001**	0.816
PID-5-BF	37.571	8.859	13.020	7.789	13.207	67.116	20.841	28.262	**< 0.001**	2.943	7.283	**< 0.001**	0.790

*CI* 95% Confidence Intervals with lower limits (*LL*) and upper limits (*UL*), *d* Cohen’s (effect size for Welch’s t-test), *r* effect size *r* for Wilcoxon rank sum test, *BDI-II* Beck Depression Inventory-II (total score), *BSCL* Brief Symptom Checklist (General Severity Index), *BSL-23* Borderline Symptom List-23 (mean score), *IPDE-Dimensional* International Personality Disorder Examination (dimensional score), *LPFS* Level of Personality Functioning Scale-Brief Form (total score), *PID-5-BF* Personality Inventory for DSM-5-Brief Form (total score); significant results in bold font

### Tasks

In a set of behavioral tasks (see Fig. 1 for an overview), participants were asked to either allocate payoffs or respond to payoff combinations for themselves and anonymous others. All interactions were one-shot exchanges, meaning participants did not engage with the same partner more than once. They were led to believe that the other people were also participants in the study. Participants were informed that they would receive a bonus based on their decisions and the decisions of other participants from the same study. That is, a few trials were drawn randomly and translated into a bonus payoff of a maximum of 5€. We chose a specific order of the tasks to minimize the influence between the tasks (see Supplementary Material section *Ordering and randomization of the tasks*).

#### SVO task

All participants performed the SVO Slider Measurement [46] which measures participants’ general social value orientation and prosocial motivation. Social value orientation delineates how individuals evaluate the allocation of resources between themselves and others and serves as a measure for active cooperation. In addition to continuous scores, the task provides a categorical classification of participants according to the primary items (altruistic, prosocial, individualistic, and competitive) and secondary items (joint gain maximizing and inequality averse).

The SVO Slider Measurement consists of 15 items (trials): six primary items to measure the general SVO score and nine secondary items to measure the prosocial motivation score. Each of the items consists of nine different prespecified joint payoff options. For example, the primary item 6 ranges from the first option with 100 points for self and 50 points for the other to the ninth option with 85 points for self and 85 points for the other (Fig. 1a or Supplementary Figure S1a). Here, a more prosocial participant would try to avoid inequality and maximize joint gain and choose option 9 (with 85 for self and 85 for the other) or one of the options close to option nine. A more individualistic participant would try to maximize their own gain and choose the first option (with 100 for self and 50 for the other) or an option close to the first option.

The secondary items can be used to differentiate between two different motivations for being prosocial: joint gain maximization and inequality aversion. For example, the secondary item 9 ranges from the first option with 100 points for self and 70 points for the other to the ninth option with 50 points for self and 100 points for the other (Supplementary Fig. 1b). Here, a joint gain maximizing participant would choose one of the options with the highest joint outcome (e.g., option 1 with 100 for self and 70 for the other), while a more inequality averse participant would choose one of the options that minimize inequality (e.g., option 4 with 81 for self and 81 for the other).

#### Dictator game

Participants played a single trial of a one-shot Dictator Game (DG; [47, 48]) in the role of the proposer and were asked to allocate 10€ between themselves and another anonymous participant as they like. Similar to the SVO task, the proposer had full control over the allocation. The allocation options ranged from 0 to 10 in steps of 1, with the amount shared serving as an indicator of active cooperation.

#### Ultimatum game

To assess reactive cooperation, participants were engaged in six one-shot UG [49] trials, each with a different anonymous proposer, in the role of the responder. The UG is similar to the DG with a key difference: The responder can either accept or reject the proposer’s offer. If the responder accepts, both players receive the proposed payoff allocation but if the responder rejects neither of them gets any money. In this context, rejection rates serve as an indicator of punishment for perceived unfair behavior and a measure for reactive cooperation [28]. In each trial, we asked participants to either accept or reject a split of 10€ offered by an anonymous participant. The six offers ranged from 1€ to 6€ in steps of 1. In line with a previous study, we considered offers from 1 to 3 as unfair and offers from 4 to 6 as fair [32].

#### Minimum acceptance rating

To examine the reliability of choices in the UG, participants played one trial of a variant of the UG [28] and were asked to indicate the minimum amount the other anonymous participant would have to offer for the participant to accept a split of 10€. Acceptance options ranged from 0 to 10 in steps of 1.

#### Fairness ratings

To test potential group differences in fairness perception, we explicitly prompted participants to assess the fairness of various hypothetical offers using the same allocation options as in the DG and UG. Offers from another anonymous participant ranged from 0 to 10€ (in steps of 1, resulting in 11 trials). Perceived fairness was rated on a 9-point Likert scale (1 = totally unfair, 9 = totally fair).

#### JPE task

All participants completed an adapted version of the Joint Payoff Evaluation (JPE) task [51]. In this task, participants rated pairs of payoffs for themselves and another anonymous participant on an 8-point Likert scale (1 = very good, 8 = very bad). Payoffs ranged from − 50 to 50 in steps of 10 (resulting in 11*11 = 121 trials). We employed a computational modeling approach to test nine different economic models, aiming to describe participants’ motives when evaluating payoff allocations.

### Questionnaire

#### Dissociality

Participants completed the German version of the Personality Inventory for ICD-11 (PiCD [45, 50]) to assess the five ICD-11 maladaptive personality domains. The PiCD is a self-report measure that consists of 60 items, with 12 items for each of the five domains: Negative Affectivity, Detachment, Dissociality, Disinhibition, and Anankastia. Each item is rated on a 5-point Likert scale (from 1 = strongly disagree to 5 = strongly agree).

### Analyses

We use the standard alpha level of *p* < 0.05 to determine whether results from the GLMM and t-tests differed significantly from those expected under the null hypothesis.

We report results from Welch’s two-sample t-test, which is considered more robust than the Student’s t-test [65]. To ensure robustness of our findings, we also conducted the Wilcoxon rank sum tests as a non-parametric alternative, which rely on ranking of observations and do not assume a specific distribution. For the experimental tasks and the dissociality factor of the PiCD questionnaire, t-tests were performed using MATLAB’s (2024) *ttest2* and *ranksum* functions. For clinical assessment, analyses were performed in R [66] using the *stats* [66] and the *coin* [67] packages. The effect sizes *Cohens’d* for Welch’s two-sample t-tests and Wilcoxon effect size *r* for Wilcoxon rank sum tests were calculated in R using the package *rstatix* [68]. For data visualization, we used R and the packages *ggplot2* [69] and *gghalves* [70].

We calculated Bayes Factors (BFs) for the tasks in which we expected no group differences to provide evidence for the null hypothesis versus the alternative hypothesis (Bayes Factor approach to equivalence testing). Bayes Factors were calculated in R [66] using the package *BayesFactor* [71] with default non-informative priors and interpreted by using the classification scheme by Jeffreys [72]. We report the BF01, which provides the posterior odds for the null hypothesis versus the alternative hypothesis and equals 1/BF10 [73].

#### SVO task

We computed the SVO score from the six primary items following the procedure described in Murphy et al. [46].

In addition, we computed the prosocial motivation score from the nine secondary items for all participants who showed consistent prosocial choices in both primary and secondary items (see Supplementary Material section *Supplementary Analyses* for details). The secondary items can be used to identify the underlying motivation for being prosocial, namely being inequality averse or aiming to maximize the joint gain.

We compared the primary SVO score between the BPD group and the healthy control group using two-sample t-tests. In addition, we calculated BFs to provide evidence for the null hypothesis versus the alternative hypothesis. For participants with consistent primary and secondary prosocial choices, we compared the secondary SVO scores between the groups using two-sample t-tests. For consistency and completeness, we provide the BFs.

#### Dictator game

We compared the amount of money allocated to the anonymous other between the groups using two-sample t-tests. In addition, we calculated BFs to provide evidence for the null hypothesis versus the alternative hypothesis.

#### Ultimatum game

We set up a Generalized Linear Mixed Model (GLMM) to test the association between rejection of offer (yes/no) as the dependent variable as well as group (BPD/HC), different offer amounts (1€, 2€, 3€, 4€, 5€, 6€), and their interaction as fixed effects and participants (subject ID) as random effects. Offer amounts were mean centered and we used the HC group as the reference category.

In addition, we set up a second GLMM to test if higher dissociality scores (PiCD [45, 50]) are related to higher rejection rates in the UG. We tested a logistic mixed model with rejection of offer (yes/no) as the dependent variable, dissociality and different offer amounts (1€, 2€, 3€, 4€, 5€, 6€) as fixed effects, and participants (subject ID) as random effect. We also tested interactions between the fixed effects variables. Offer amounts and dissociality scores were mean centered. Note that the models differ from our preregistered models due to incorrect specifications in the preregistration.

The analysis was performed with the MATLAB function *fitglme.* We specified our models with a binomial response distribution and a logit link function and used MATLAB’s maximum pseudo likelihood (MPL) estimation to fit the models. We calculated Odds Ratios (OR) by exponentiating the coefficients.

#### Minimum acceptance rating

We tested the smallest accepted offer between the groups using two-sample t-tests.

#### Fairness ratings

We set up a GLMM to test the association between perceived fairness (ratings from 1 = totally unfair, 9 = totally fair) as the dependent variable, group (BPD/HC), different offer amounts (0€ to 10€), and their interaction as fixed effects, and participants (subject ID) as random effect. Offer amounts were mean centered and included as a quadratic term. The analysis was performed with the MATLAB function *fitglme.* We specified our models with a normal response distribution, an identity link function and used MATLAB’s MPL estimation to fit the models with the HC group as the reference category.

#### JPE task

We compared nine different economic models to test participants’ social motives in evaluating the payoff pairs. Each participant’s evaluation during the JPE task was linearly regressed with different predictors of the specified economic models. See Supplementary Material section Supplementary *Analyses* for model descriptions.

To assess model evidence, we conducted both fixed-effects analyses, using log-group Bayes Factors, and random-effects analyses, using protected exceedance probabilities (see Supplementary Material section *Supplementary Analyses* for details).

We postulated that the Fehr-Schmidt model, which considers own gain, disadvantageous inequality, and advantageous inequality, would provide the best fit for both group’s data and that individuals from the BPD group would show more inequality aversion. Therefore, we tested if the BPD group shows higher weights on the disadvantageous inequality aversion parameter compared to the control group. We conducted two-sample t-tests to compare model parameter weights between the groups.

#### Mini meta-analysis on ultimatum games

The meta-analysis was conducted in R [66] using the *meta* package [74], following the guidelines outlined by Harrer et al. [75]. For each study, between-group effect sizes (standardized mean differences) were calculated as Cohen’s d. For the study reporting BPD feature scores, participants were divided into groups based on established cut-off criteria [76]. If studies reported outcomes separately for additional conditions, the data were aggregated across these conditions before calculating the effect sizes. Detailed information about the study selection, analyses, and characteristics of the included studies are provided in the Supplementary Material section *Mini Meta-Analysis on Ultimatum Games*.

## Results

### SVO task

#### General social value orientation (H1)

We employed the SVO measurement to test hypothesis H1 that individuals with BPD and controls do not differ in their general social value orientation and willingness to actively cooperate in the primary items of the SVO task.

The majority of participants in both groups was categorized as prosocial (BPD = 89.66%, HC = 98%), with the remaining few categorized as individualistic (BPD = 10.34, %, HC = 2%).

**Fig. 2:**
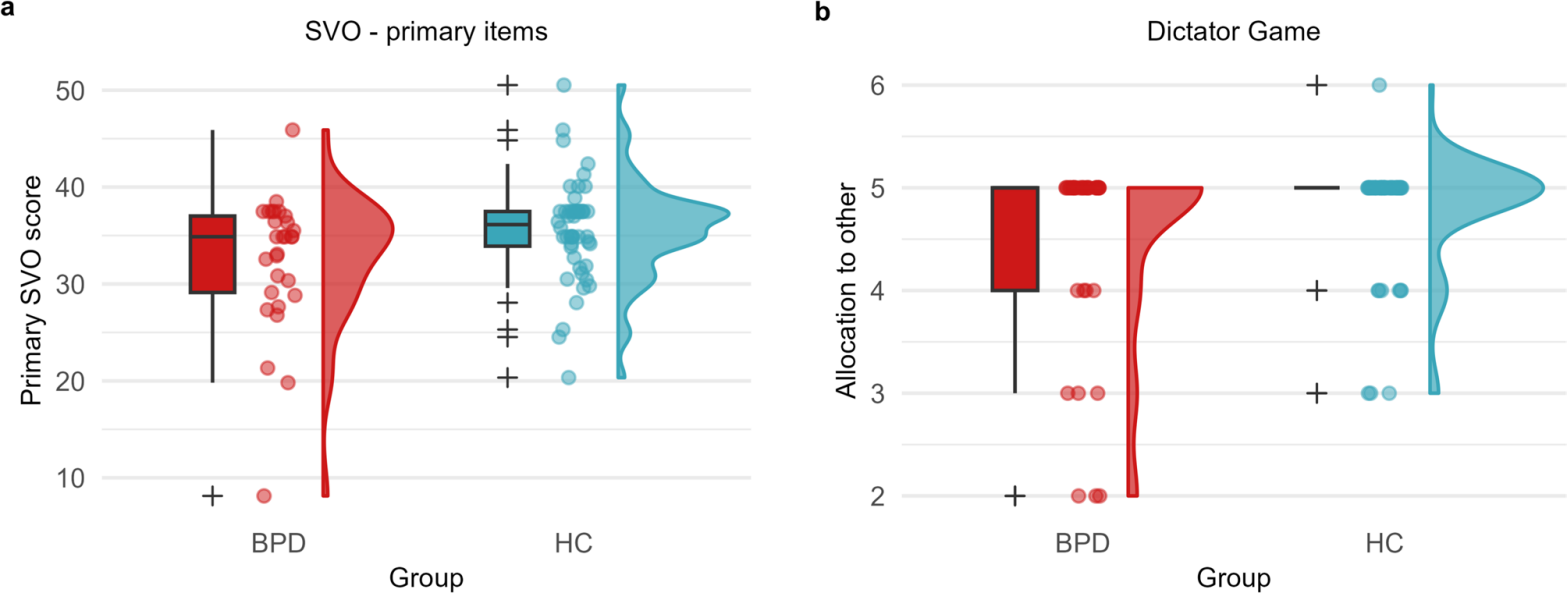
Active cooperation task results. Raincloud plots show the distribution of (**a**) SVO scores (primary items) and (**b**) the distribution of allocations of 10€ to the anonymous partner in the BPD group and the HC group. The plot includes individual scores for each participant, a box plot, and a probability density plot. The boxplot visualizes the median, the 25th and 75th percentiles, two whiskers (extending from the upper and lower hinges to the largest and lowest value within 1.5 times the inter-quartile range, and all outliers individually. The density plot was generated using Gaussian kernel density estimation

When analyzing the continuous SVO scores, we found a significant difference between the BPD group and the control group with the control group demonstrating higher, and therefore more prosocial scores (Table 4; Fig. 2a). The Bayes Factor analysis revealed a BF01 of 0.47, indicating only anecdotal evidence for the absence of an effect [72, 77]. While continuous SVO scores indicated a potential group difference, the Bayesian analysis did not provide strong evidence for neither the null hypothesis nor the alternative hypothesis. Given that the vast majority of participants in both groups were categorized as prosocial, we cannot draw definitive conclusions regarding differences in active cooperation between individuals with BPD and healthy controls.

**Table 4:** Summary of statistical results comparing the BPD and HC group

Task	Hypothesis	BPD			HC			Welch’s t-test						Wilcoxon rank sum test		
		*M*	*SD*	*N*	*M*	*SD*	*N*	*t*	*DF*	*CI (LL)*	*CI (UL)*	*p*	*d*	*z*	*p*	*r*
SVO (G)	H1	32.421	7.127	29	35.579	5.203	50	−2.085	45.497	−6.207	−0.109	**0.043**	−0.506	−1.989	**0.047**	0.224
SVO (PM)	H2	0.264	0.101	24	0.287	0.211	44	−0.592	65.242	−0.098	0.053	0.556	−0.136	0.225	0.822	0.028
DG	H3	4.457	0.980	35	4.780	0.582	50	−1.745	50.683	−0.694	0.049	0.087	−0.400	−1.456	0.145	0.159
UG	H4	36.190	23.391	35	40.476	26.571	49	−0.782	78.389	−0.152	0.066	0.437	−0.717	−0.746	0.455	0.082
MA	H5	4.057	2.222	35	4.280	1.852	50	−0.487	64.520	−1.138	0.692	0.628	−0.109	−0.279	0.780	0.031
JPE	H7.2	0.023	0.029	35	0.025	0.026	50	−0.367	69.104	−0.015	0.010	0.715	−0.082	−0.246	0.806	0.027

This table reports results from task-based comparisons between the BPD and HC groups using Welch’s t-tests and Wilcoxon rank sum tests. Task: abbreviated name of the experimental task (and measure); Hypothesis: preregistered hypothesis; *CI* 95% Confidence Intervals with lower limits (*LL*) and upper limits (*UL*), *d* Cohen’s (effect size for Welch’s t-test), *r* effect size *r* for Wilcoxon rank sum test; Task abbreviations and dependent variables measured: *SVO* Social Value Orientation (G = general SVO score measured with the primary items, *PM* Prosocial Motivation score measured with the secondary items), *DG* Dictator Game (allocation to the other), *UG* Ultimatum Game (rejection rates), *MA* Minimum Acceptance rating (minimum offer required to accept the split), *JPE* Joint Payoff Evaluation task (disadvantageous inequality aversion parameter weights of the Fehr-Schmidt model); significant results in bold font

#### Prosocial motivation (H2)

To differentiate between two different motivations for being prosocial (joint gain maximization and inequality aversion) and test hypothesis H2 that individuals with BPD are more inequality averse than healthy controls, we analyzed the secondary items of the SVO measurement.

Among the prosocial participants, all in the BPD group were categorized as inequality averse, as were the majority in the control group (inequality averse N_*Hc*_ = 88.64%, joint gain maximizing N_*Hc*_ = 11.36%; Supplementary Figure S2). We did not find a significant difference in prosocial motivation scores between the groups (Table 4). For consistency, we calculated Bayes Factors. The analysis revealed a BF01 of 3.49, indicating that the data were approximately 3.5 times more likely under the null hypothesis than under the alternative hypothesis (BF10 = 0.29). This provides moderate evidence for the absence of an effect [72]. These results suggest no difference in inequality aversion (versus joint gain maximization) in individuals with BPD compared to healthy controls.

### Dictator game (H3)

To test Hypothesis H3—that the groups do not differ in active cooperation in the DG—we compared the amount of money allocated to another person in the DG. The results showed that participants with BPD shared a similar amount to participants from the control group, with no significant difference observed (Table 4; Fig. 2b). We conclude that participants from both groups exhibited equally cooperative behavior in the DG. The Bayes Factor analysis revealed a BF01 of 0.92, demonstrating only anecdotal evidence in favor of the null hypothesis [72, 77].

### Ultimatum game (H4)

We used the UG to test H4 that individuals with BPD, compared to individuals without BPD show impairments in reactive cooperation and therefore higher rejection rates. Across all possible offers, the mean rejection rates did not differ significantly between the groups (Table 4).

**Fig. 3:**
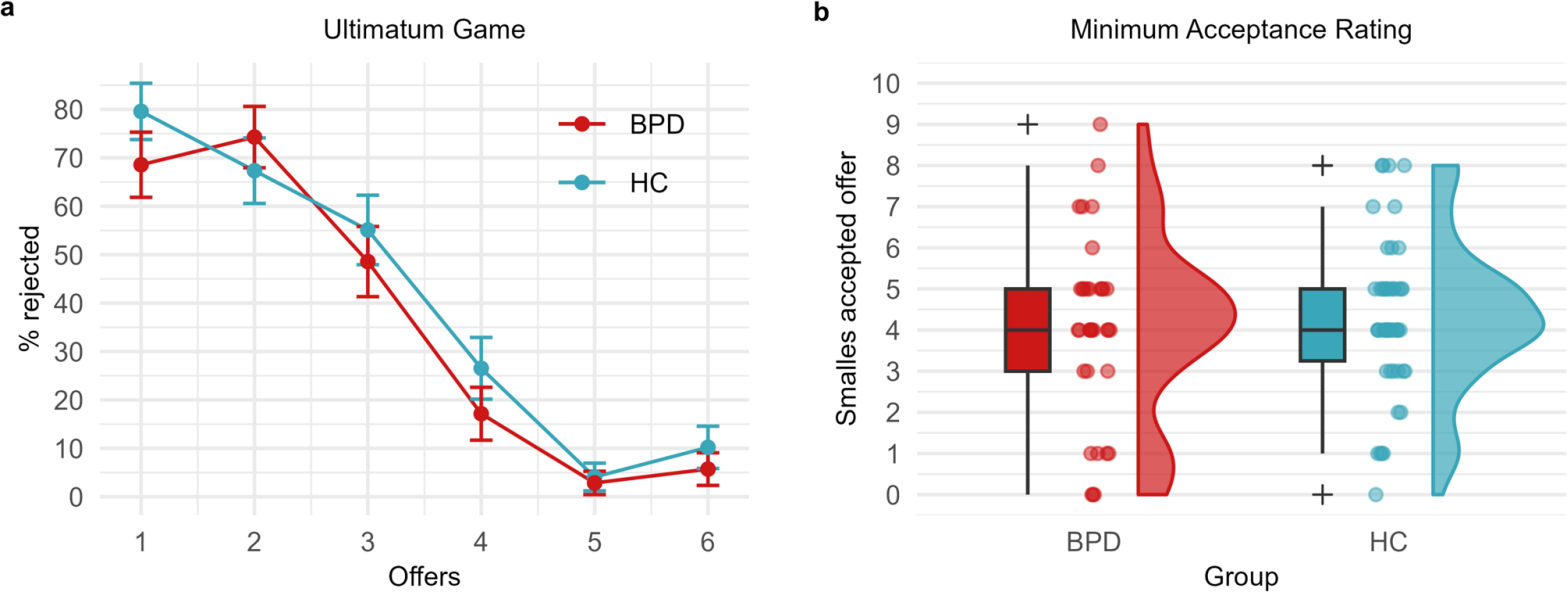
Reactive cooperation task results. **a** Mean rejection rates for each possible offer in the UG in the BPD group and the HC group. Error bars show the standard error of the mean (SEM). **b** Raincloud plots show the distribution of the smallest accepted offers in the Minimum Acceptance Rating in the BPD group and the HC group

We set up a GLMM to test the association between rejection of offer (yes/no) and group (BPD/HC), different offer amounts (1€, 2€, 3€, 4€, 5€, 6€), and the interaction between these factors. We included the data of 35 BPD participants and 49 HC participants in this analysis. The results indicated that for the HC group (reference) the *Offer* amounts significantly predicted rejection rates (*b* = −1.14, *SE* = 0.127, *t*_(500)_ = −8,971, *p* < 0.001, 95% *CI* [−1.389, −0.89], *OR* = 0.32). The *Group* (*b* = −0.335, *SE* = 0.456, t_(500)_ = −0.736, *p* = 0.462, 95% *CI* [−1.231, 0.56], *OR* = 0.715) and *Group x Offer* interaction (*b* = −0.007, *SE* = 0.2, t_(500)_ = −0.037, *p* = 0.971, 95% *CI* [−0.4, 0.386], *OR* = 0.993) were not significant predictors. These results suggest that the rejection rates seem to depend solely on the offer amounts and that there is no reduction in reactive cooperation in the BPD group compared to the control group (see Fig. 3a).

### Dissociality (H8)

Taking a dimensional approach, we investigated whether the maladaptive trait of dissociality is associated with reduced reactive cooperation as specified in hypothesis H8. We specified a second GLMM to test the association between rejection of offer (yes/no) and different offer amounts (1€, 2€, 3€, 4€, 5€, 6€), dissociality scores, and the interaction between these factors. We included the data of 35 BPD participants and 49 HC participants in this analysis. Again, the results indicated that *Offer* significantly predicted rejection (b = −1.142, SE = 0.098, t_(500)_ = −11.644, *p* < 0.001, 95% CI [−1.335, −0.949], *OR* = 0.319). The effect of *Dissociality* (b = 0.002, SE = 0.027, t_(500)_ = 0.089, *p* = 0.929, 95% CI [−0.051, 0.056]. *OR* = 1.002) and *Offer* x *Dissociality* interaction (b = 0.003, SE = 0.012, t_(500)_ = 0.25, *p* = 0.803, 95% CI [−0.02, 0.026], *OR* = 1.003) were not significant predictors. We conclude that higher dissociality scores are not related to higher rejection rates in the UG. We report group mean comparisons for dissociality scores and descriptive statistics in the Supplementary Material section *Supplementary Results* and Supplementary Figure S3).

### Minimum acceptance rating (H5)

To test H5—that individuals with BPD show impaired reactive cooperation and therefore require higher offers to accept an allocation compared to controls—and to examine the reliability of choices in the UG, we tested for differences in the smallest accepted offers between the groups. The results showed that participants with BPD did not require a higher offer compared to participants from the control group (Table 4; Fig. 3b) to accept.

### Fairness ratings (H6)

**Fig. 4:**
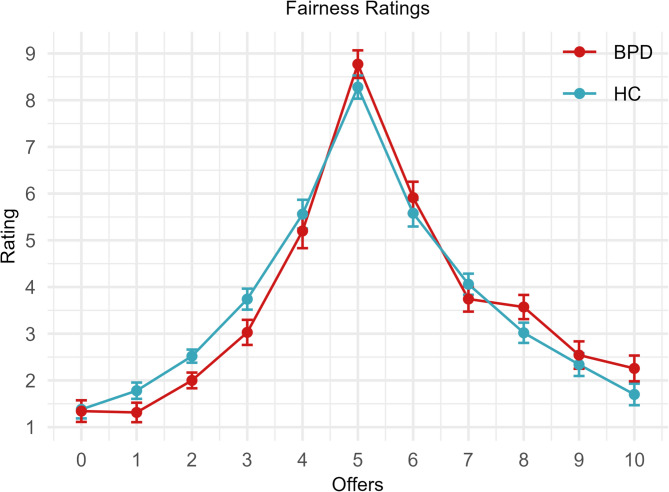
Mean ratings of fairness for each possible split of 10€ (with steps of 1€) in the BPD group and the HC group. Error bars show the standard error of the mean (SEM). In both groups, offers were perceived as increasingly fair up to the offer of 5€ and perceived as less fair for offers higher than 5 (significant quadratic effect in GLMM). We found no significant group differences in the quadratic effect of offers on fairness perception

To test H6—that the BPD group would judge fair offers as less fair than the HC group—we set up a GLMM with linear and quadratic terms to examine the association between perceived fairness and group, different offer amounts, and their interaction (see Table 5 for the full model summary). For the HC group (reference), there was a significant positive linear effect of *Offer* and a significant negative quadratic effect, suggesting an inverted U-shaped relationship between offer amounts and fairness ratings. A significant linear *Group* x *Offer* interaction revealed that the BPD group exhibited a steeper slope at the mean offer level (offer = 5) compared to the HC group. Importantly, the linear interaction reflects group differences in the slopes at the center of the offer range, not a consistent group difference across all offer levels, as the slopes change across the offer range due to the quadratic component. The *Group* x quadratic *Offer* interaction was not significant, suggesting that the quadratic relationship between offer and fairness rating was similar across groups. Together, these results indicate that offers are perceived as increasingly fair up to a certain value, after which higher offers are perceived as less fair by both groups (see Fig. 4 for group-wise fairness ratings across offers). We report descriptive statistics for disadvantageous offers (offer 0 to 3), fair offers (4 to 6), and advantageous offers (7 to 10) for both groups in the Supplementary Table S2.

**Table 5:** Model summary of the GLMM on the linear and quadratic effects of group, offer, and their interaction on fairness ratings

Predictors	b	SE	t	DF	*p*	CI (LL)	CI (UL)	OR
(Intercept)	5.550	0.151	36.801	929	**< 0.001**	5.254	5.846	257.172
Group [BPD]	−0.114	0.235	−0.487	929	0.626	−0.576	0.347	0.892
Offer	0.055	0.026	2.100	929	**0.036**	0.004	0.106	1.056
Group [BPD] x Offer	0.094	0.040	2.323	929	**0.020**	0.015	0.173	1.099
Offer^2^	−0.192	0.009	−20.613	929	**< 0.001**	−0.210	−0.173	0.826
Group [BPD] x Offer^2^	0.009	0.014	0.618	929	0.537	−0.019	0.037	1.009

*CI* 95% Confidence Interval *LL* Lower Limit, *UL* Upper Limit; significant results in bold font; Sample size: N_BPD_ = 35, N_HC_ = 50

### JPE task (H7)

To explore participants’ motives when evaluating payoff allocations, we tested how well nine different economic models fit their evaluations of allocation pairs. We included the data of 35 BPD participants and 49 HC participants in this analysis. As hypothesized in H7.1, the fixed-effects analysis based on log-group Bayes Factors revealed that the Fehr-Schmidt model, which accounts for payoffs for self, disadvantageous, and advantageous inequality, was the best fitting model in both groups. We performed an additional random-effects analysis using protected exceedance probabilities and found that the Inequality & Joint-Gain model was the best-fitting model in both groups (Supplementary Figure S4). The inequality & Joint-Gain model is also the second-best model in both groups according to the log-group Bayes Factors. This implies that the Inequality & Joint-Gain model performed better in more participants in both groups. For those participants, for whom the Fehr-Schmidt model performed better, it did so by a larger margin. To test H7.2—that individuals with BPD show heightened inequality aversion compared to controls—we compared disadvantageous inequality parameter weights of the Fehr-Schmidt model. We found no significant difference in the disadvantageous inequality parameter weights between the groups (Table 4). In conclusion, inequality and gain magnitude are important factors for participants in both groups when evaluating joint payoffs, and there is no evidence of heightened inequality aversion in the BPD group compared to the control group.

### Mini meta-analysis on ultimatum games

**Fig. 5:**
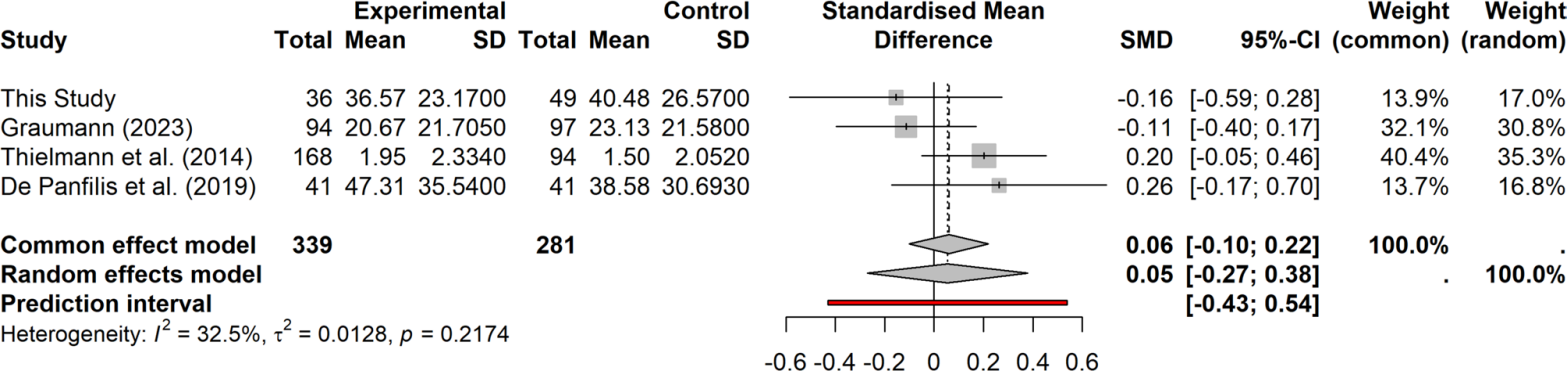
Mini meta-analysis. Forest plot comparison of studies evaluating rejection rates in the Ultimatum Game in individuals with BPD (features) and controls. The standardized mean difference (SMD) for each study is displayed with the line representing the confidence interval and the square representing the weight in the analysis. The diamond symbolizes the average effect, its length represents the confidence interval. Experimental = BPD group, Control = control group, CI = confidence interval

We included four studies that met our inclusion criteria (see Supplementary Material section *Mini Meta-Analysis on Ultimatum Games* for details and Supplementary Figure S5 and Supplementary Table S3) in our meta-analysis and found an overall effect size of *d* = 0.054 (95% CI [− 0.271, 0.379]), indicating a small, non-significant effect (*p* = 0.633). Heterogeneity across studies was low (*I²* = 32.5%, 95% *CI* [0.0, 76]; *τ²* = 0.013, 95% *CI* [0.00, 0.599]), suggesting minimal variability in effect sizes (see forest plot, Fig. 5). Therefore, no significant overall effect of group membership on rejection rates in the Ultimatum Game was found. A funnel plot (see Supplementary Figure S6) revealed no indication of publication bias.

## Discussion

In this study, we aimed to explore the mechanisms underlying social dysfunctions in individuals with BPD by examining their social preferences and behaviors in a comprehensive set of standard economic games. While we hypothesized no differences in active cooperation, we expected lower reactive cooperation and altered fairness perception and sensitivity to inequality in the BPD group. However, when tested in anonymous, one-shot economic games individuals with BPD did not differ from healthy controls in any of the tasks. Our study was strengthened by using multiple tasks within the same well-characterized sample, a preregistered design, and the application of robust and elaborate statistical analyses to ensure rigorous examination of the data. In the following, we discuss active, reactive cooperation, fairness perception and inequality aversion in turn.

### Active cooperation

With respect to active cooperation as measured by social preferences, we expected no differences between participants with and without BPD. We found a small but significant difference in continuous SVO scores, namely participants in the control group demonstrated higher SVO scores. However, this result should be interpreted with caution. The average SVO score for the BPD group was close to the SVO scores of the BPD and control samples in a previous study that showed no group differences [10]. Our control group exhibited higher SVO scores than both the BPD and the control group in Lévay et al. [10] and also higher scores than we found in three healthy samples in our previous work [78]. Despite the significant difference between the groups, the average group scores of both groups still fall within the prosocial category. This suggests that the significant difference between the groups may be driven by exceptionally high prosocial preferences in the control group, rather than a low prosocial preference in the BPD group. The Bayes Factor analysis revealed that no clear distinction between the null and the alternative hypothesis is possible. Thus, even though we found significantly lower SVO scores in the BPD group compared to the control group, we cannot conclude that people with BPD are impaired in active cooperation.

With respect to active cooperation as measured by the DG, we found no group differences. Participants in the BPD group shared a similar amount of money compared to the control group. This result is consistent with our hypothesis and previous studies that reported no differences between BPD and HC groups in investment in a DG with real incentives and anonymous partners [29, 31, 43]. Similarly, Thielmann et al. [28] reported that they found no association between BP features and hypothetical offers in a DG in a large community sample (*N* = 556), suggesting that high levels of BP features are not related to impairments in active cooperation. Our results are also in line with a study reporting no group differences in the first round of investment in a TG between a BPD and a healthy control group [17].

Taken together, our results replicate previous studies and suggest that people with BPD show intact prosocial preferences and no impairment in active cooperation (i.e., non-exploitation) [28].

### Reactive cooperation

Next, we tested reactive cooperation to determine whether previous findings on impaired reactive cooperation (i.e., non-retaliation) in people with high levels of BP features would also extend to patients diagnosed with BPD [28]. We therefore implemented an UG in which participants played the role of the receiver and were able to retaliate if they found the proposer’s offer unfair. We tested if BPD (effect of group) or the maladaptive personality trait dissociality significantly affected rejection rates in the UG. Participants in both groups accepted higher offers at a higher rate and lower offers at a lower rate and there was no significant difference between the groups. Therefore, neither BPD diagnosis nor dissociality scores had an effect on reactive cooperation. To test the robustness of the UG results, we asked participants to indicate the lowest offer they would be willing to accept if a proposer were to share 10€. We found no significant differences between the BPD and control groups regarding minimum acceptance ratings, which suggests no indication of less reactive cooperation in the BPD group. This finding is consistent with our UG results and indicates that the threshold for acceptable offers does not significantly differ between the groups.

Previous studies on BPD and the UG have reported mixed findings. Polgár et al. [79] tested 47 BPD patients and 43 healthy controls in a 40-trial multi-round UG. BPD participants accepted more unfair offers than controls, and while positive facial expressions increased unfair offer acceptance among controls, they had no effect on those with BPD. De Panfilis et al. [32] tested 41 BPD patients and 41 controls in a UG with emotion regulation strategies. BPD participants rejected fair offers more frequently (independent of the emotion regulation strategy used) and perceived them as less fair, suggesting a bias toward underestimating positive feedback. Thielmann et al. [28] examined 559 participants with varying levels of BP features. In a one-shot strategy variant, those with higher BP features demanded higher offers, while in a game variant, they rejected more offers after a breakdown in cooperation.

Our results do not align with previous findings of either lower rejection rates [79] or higher rejection rates in individuals with BPD [32] or elevated BP features [28]. Differences in study design, such as the inclusion of emotion regulation strategies [32], the manipulation of facial expressions [79], or the use of BPD features instead of clinical diagnosis as criterion [28], could have contributed to the variability in outcomes. To assess these differences in the literature, we conducted a mini meta-analysis as part of the present study. The results showed no significant group differences, with small heterogeneity of effect sizes and no indication of publication bias. These findings suggest that BPD-related differences in the UG may not generalize across variations in task design.

Overall, our findings indicate that individuals with BPD are just as capable of forgiving uncooperative behavior as healthy controls. While our results diverge from previous findings using UG paradigms, they are consistent with studies employing multi-round TG paradigms, which have demonstrated that individuals with BPD can show forgiveness in response to unfair behavior [17, 30]. For future research, we recommend the use of more ecologically valid tasks involving real social interactions [3, 43] that are relevant to BPD symptomatology.

### Fairness perception and inequality aversion

Regarding fairness perception and sensitivity to inequality, prior research indicates that individuals with BPD often feel excluded, even when included or overincluded [15, 18, 80], perceive fair exchanges as less fair and underestimate positive feedback [25, 32], and exhibit high victim sensitivity [37]. They also tend to punish fair behavior more frequently [15] and struggle to rebuild cooperation after being treated unfairly [25]. Based on these findings, we expected individuals with BPD to rate fair offers as less fair and show greater inequality aversion than controls.

Contrary to our hypothesis, we found no significant group differences in fairness perception in our standard task. These results are consistent with participants’ UG behavior and minimum acceptance ratings, suggesting that individuals with BPD do value equality and fairness and do not differ significantly in related behaviors from healthy controls in these economic games. Our results also align with those of Jeung et al. [41], who found that fairness perception remains intact in individuals with BPD. Notably, half of the BPD group in their study chose an unfair interaction partner, which the authors suggest may be linked to BPD patients’ negative and instable self-image, contributing to dysfunctional social interactions and relationships [41].

We compared several economic models on participants’ evaluations of a broader range of payoff pairs to better understand the mechanisms governing social preferences. Here, we used a large payoff space including negative payoffs. We found that two related models performed well across both samples: the Fehr-Schmidt model and the Inequality & Joint-Gain model, which is in line with the results from previous work in healthy samples [78]. Both models include metrics for inequality and joint- or self-gain. When looking at disadvantageous inequality model parameter weights (Fehr-Schmidt model), we found no differences between the groups. This suggests that for both groups inequality and gain magnitude play an important role when evaluating joint payoffs and there seems to be no heightened inequality aversion in individuals with BPD compared to individuals in the control group. Additionally, we found that all participants in the BPD group and the majority of the HC group were categorized as inequality averse based on secondary SVO scores. This indicates that in both groups high sensitivity to inequality may be a central driver for prosocial behavior.

In summary, our findings indicate that both individuals with BPD and healthy controls exhibit inequality aversion and value fairness. Although these results were unexpected, they are consistent with prior research suggesting that individuals with clinically relevant BPD features may display heightened justice concerns not only for themselves but also for others [37]. Supporting this, Wischniewski and Brüne [42] found that while individuals with BPD punished unfair behavior in a modified DG to the same extent as controls, their motivation differed: whereas control participants were primarily driven by self-interest, the BPD group appeared motivated by angry retaliation, likely due to identifying with the victim’s perspective. Our findings also align with those of previous studies reporting no differences between individuals with BPD and controls in fairness appraisals of trustees in TG paradigms [30, 40].

### Strengths, limitations, and outlooks

In this study, we used a set of standard economic tasks to assess baseline levels of social preferences, active and reactive cooperation, fairness perception and inequality aversion in women with and without BPD. By deliberately avoiding additional experimental conditions, we sought to minimize confounding factors, which, however, entails limitations. We here discuss four specific limitations and make suggestions for future studies.

First, the lack of direct social interactions and variations in social contexts in our design may have limited the possibility to dissect more nuanced group differences. Participants made hypothetical choices or believed their partner was an anonymous participant. While anonymity in economic games can reveal underlying preferences by removing external influences like concerns about reputation or consequences [81], the absence of direct interaction may have reduced the tasks’ relevance, particularly for BPD-related behaviors. Including contextual factors like facial expressions or emotion regulation strategies could have made these tasks more salient. Nonetheless, we emphasize the importance of using standard economic games to establish baseline behavior before introducing task modifications to test specific questions [81]. Ideally, studies should start with baseline assessments using standard economic tasks, followed by specific alterations combined with self-reports and real social interactions. Future research should incorporate direct social interactions in repeated game paradigms to better simulate real-world social dynamics [3, 82] or test participants in naturalistic approaches with close others [83]. Moreover, the use of emotion induction procedures, as suggested by Jeung et al. [22], may help clarify how emotional states influence social decision-making in BPD. For instance, Masland and Hooley [84] found that negative emotional primes specifically influenced trustworthiness appraisals in individuals with higher levels of BPD symptoms. An especially important direction for future studies is the consideration of situational context. Prior research suggests that impairments in social behavior among individuals with BPD may be particularly pronounced following unexpected positive interactions or feedback [17, 30, 32, 33]. Differentiating responses to positive versus negative social contexts may be key to understanding the variability and triggers of social dysfunction in BPD. We specifically recommend that future studies adopt multi-method approaches—integrating self-report, behavioral, and physiological measures—to assess baseline behavior across diverse social contexts. For instance, the same economic game could be administered with different types of social partners—such as virtual agents, unfamiliar strangers, or close others—to investigate how relationship context shapes the association between maladaptive personality traits and reactive cooperation in individuals with BPD. Additionally, the impact of situational context could be explored by comparing behavior in these games following positive versus negative interaction scenarios [17].

Second, the use of one-shot games may not fully capture the complexities of repeated social interactions. Multi-round games in previous research have repeatedly revealed alterations in individuals with BPD [25, 26, 79], especially in reactive cooperation, which may emerge over time. For example, Abramov et al. [30] emphasized the importance of capturing the dynamic nature of social behavior by using a multi-round TG. Their findings revealed paradoxical patterns among individuals with higher BPD trait counts, whose behavior shifted across different phases of the game. These results highlight the need to investigate how trust and cooperation develop over time—an approach that may be particularly crucial for understanding reactive forms of social behavior.

Third, our study did not incorporate personality measures based on the HEXACO model [85], particularly the honesty-humility and agreeableness dimensions, which have been shown to play distinct roles in active and reactive cooperation [28]. Active cooperation—which involves non-exploitation and a willingness to share or give, is tied to honesty-humility, while reactive cooperation—which refers to non-retaliation and the capacity to forgive after unfair treatment—is associated with HEXACO agreeableness [35, 86]. Borderline personality features are related to deficits in reactive cooperation due to low levels of HEXACO agreeableness, yet these features do not appear to impair active cooperation [28, 34]. Although the HEXACO and Big Five models share some conceptual overlap, they differ in key aspects: HEXACO honesty-humility captures traits such as fairness, sincerity, and greed-avoidance, whereas HEXACO agreeableness emphasizes patience, forgiveness, and non-retaliation [85]. In comparison, Big Five agreeableness reflects trust, altruism, and compliance [87]. Given these distinctions, they do not represent the same underlying personality constructs and cannot be used interchangeably [88]. Future research should therefore incorporate HEXACO personality assessments when examining cooperative behavior of individuals with BPD under varying situational affordances [44]. This line of research further underscores the value of adopting dimensional approaches for understanding psychopathology. In our study, we aimed to address this by including a measure of maladaptive personality traits. We advocate for the continued integration of dimensional approaches alongside traditional categorical models, as this combination allows for a more comprehensive understanding of impairments and behavioral patterns while remaining applicable in clinical settings—particularly in the context of personality disorders [89]. Dimensional frameworks, such as the Research Domain Criteria (RDoC; [90]) initiative, advocate for investigating mental health through core functional domains rather than relying solely on categorical diagnostic labels. This shift enables a more nuanced understanding of individual differences and the underlying mechanisms that contribute to social dysfunction in BPD.

Lastly, our sample is limited in terms of gender and size. We only tested female participants as many studies on BPD [22] and our sample size may have reduced the statistical power to detect subtle group differences. Nonetheless, we want to stress that our study has a relatively large and well-characterized sample of BPD patients, matched with controls on age and intelligence. Achieving adequate sample sizes remains a broader challenge in psychological and clinical research, where sample sizes are often determined by convention rather than power analyses [91]. This issue is further complicated by the high behavioral variability in BPD populations and the potentially small effect sizes observed in low-emotion economic tasks. We underscore the importance of reporting effect sizes, justifying sample sizes [92], and leveraging existing datasets. In our study, we addressed this challenge by integrating existing data and performing a mini meta-analysis for the UG. We advocate for the use of similar strategies—drawing on available datasets and using meta-analytic procedures to summarize comparable studies [52].

Despite these limitations, our study provides valuable insights and underscores the importance of further research with robust and transparent methodologies to better understand social behavior in individuals with BPD. Replications with larger, more diverse samples will be essential to confirm and extend our findings. Looking ahead, preregistration of hypotheses—as encouraged by open science platforms such as the Open Science Framework [93] or AsPredicted [94]–can help promoting more reliable results. In line with these recommendations, we preregistered our study and have made our behavioral data and analysis code openly available. Reporting null results and sharing data and code are critical steps toward enhancing transparency and reproducibility. These practices not only strengthen the credibility of individual studies but also contribute to addressing the broader replication crisis in clinical psychology and psychiatry [95]. Broader adoption of open science initiatives and active participation in collaborative consortia—such as the German Reproducibility Network [96], or the German Center for Mental Health (DZPG; [97])—can facilitate open large-scale, multi-site studies that enhance the generalizability and impact of clinical research.

## Summary/conclusion

Our study did not find significant differences in active or reactive cooperation between individuals with BPD and healthy controls. These results suggest that, at least in the context of anonymous one-shot games, cooperation seems not to differ between those with BPD and healthy individuals. This underscores the importance of considering fine-grained contextual factors of social interactions for understanding cooperative decision-making in BPD.

Understanding the mechanisms of social dysfunction in BPD is crucial for developing therapeutic interventions that can help individuals form stable and successful relationships. By exploring the specific contexts and underlying reasons for these dysfunctional interactions, we can better tailor interventions to address these challenges, ultimately improving social functioning and quality of life for those with BPD.

In conclusion, our study provides valuable insights into the social decision-making processes of individuals with BPD. We emphasize the role of context and the need for replication. Further research is needed to refine these findings and to develop targeted interventions that can help individuals with BPD improve their social interactions and relationships.

## Supplementary Information

Supplementary Material 1.

## Data Availability

The data and code for this study are publicly available on Github (https://github.com/dnhi-lab/CooperativeDecisionsBPD_2025) and Zenodo (https://zenodo.org/records/15322897).

## Abbreviations

BFs: Bayes Factors
BP: Borderline Personality
BPD: Borderline Personality Disorder
DG: Dictator Game
GLMM: Generalized Linear Mixed Model
HC: Healthy Control
IPDE: International Personality Disorder Examination
JPE: Joint Payoff Evaluation
Mini-DIPS: short version of the Diagnostic Interview for Mental Disorders
Mini-q: three-minute speeded reasoning test
MPL: Maximum Pseudo Likelihood
OR: Odds Ratios
PiCD: Personality Inventory for ICD-11
SVO: Social Value Orientation
TG: Trust Game
UG: Ultimatum Game
