# Supplementary material for: Cooperative decision-making in borderline personality disorder: insights from a preregistered study using a comprehensive economic task battery
Source: Borderline Personal Disord Emot Dysregul. 2025 Jun 17;12:24. doi: 10.1186/s40479-025-00295-2 (PMC12172245; doi:10.1186/s40479-025-00295-2)
Supplement: Supplementary file 1 — Supplementary Material 1. [file 40479_2025_295_MOESM1_ESM.docx]

# Supplementary Material

## List of Interviews and Questionnaires

**Supplementary Table S1.** Interviews and Questionnaires

| Name | Type |
| --- | --- |
| Mini-DIPS Open Access (German version of the Anxiety Disorders Interview Schedule; 1) | Interview |
| IPDE (International Personality Disorder Examination; 2) | Interview |
| BSL-23 (Borderline Symptom List-23; 3) | Self-report |
| BSCL (Brief-Symptom-Checklist; 4) | Self-report |
| BDI-II (Beck Depression Inventory; 5) | Self-report |
| ASRS-v1.1 (Adult ADHD Self-Report Scale; 6) | Self-report |
| ADHS-SB (ADHS-Self-Report for adults; 7) | Self-report |
| WURS-K (German version of the Wender Utah Rating Scale; 8) | Self-report |
| MDBF (Multidimensional Mood Questionnaire – Short Version A; 9) | Self-report |
| NEO-FFI (German version; 10) | Self-report |
| PID-5-BF (German version of the Personality Inventory for DSM-5-Brief Form; 11,12) | Self-report |
| STAXI-2 (German Version of the State-Trait Anger Expression Inventory-2; 13) | Self-report |
| BIS-11 (German Version of the Barratt Impulsiveness Scale; 14) | Self-report |
| IERQ (Interpersonal Emotion Regulation Questionnaire; 15,16) | Self-report |
| mini-q (A Three-Minute Intelligence Screening; 17) | Self-report |
| PiCD (The Personality Inventory for ICD-11; 18,19) | Self-report |
| LPFS-BF (Level of Personality Functioning Scale-Brief Form; 20,21) | Self-report |
| IES-27 (Impulsivity and Emotion Dysregulation Scale for Borderline Personality Disorder; 22) | Self-report |

## Supplementary Methods

### Participants

#### Power analysis

We performed a power analysis using G*Power version 3.1.9.2 (23) for sample size estimation, based on data from a previous study (24) on group differences in updating for undesirable feedback in BPD patients (N = 22) and healthy controls (N = 42). The effect size in Korn et al. (24) was d = 0.65. With an alpha criterion of .05 and a power of 1-β = .80, the minimum sample size needed is N = 30 for the patients’ group and N = 30 for the control group for a one-tailed two-sample t-test.

#### Inclusion and exclusion procedure

We excluded individuals with severe neurological disorders or head/brain injuries, severe physical illness, BMI < 15, severe visual impairments, cognitive disorders, lifetime diagnosis of schizophrenia, schizoaffective or bipolar disorder, or acute suicidality from participation. Additionally, psychology students, except those in their first semester, were not included.

Participants with BPD had to meet four or more BPD criteria according to the IPDE interview to be included (i.e., we also included participants with sub-threshold BPD who met less than the threshold of 5 criteria but had a prior diagnosis). For the BPD group further exclusion criteria included current diagnosis of narcissistic or avoidant personality disorder, current moderate or severe alcohol or drug abuse, and the use of psychotropic medication, except for antidepressants, anxiolytic medication, on-demand medication, and limited Quetiapin prescriptions for sleep efficiency (up to 100 mg retarded and 50 mg non-retarded). Control group participants were also subject to additional exclusion criteria, including current alcohol or drug abuse, lifetime diagnosis of personality disorders, other psychiatric diagnoses (within the last 5 years), recent psychological or psychiatric interventions (within the last 5 years), presence of more than one current BPD symptoms (according to the IPDE), current clinically relevant symptoms ( assessed via the Mini-DIPS, 1), and the use of psychotropic medication.

We planned to collect 30 valid datasets per group (i.e., datasets that fulfill our preregistered inclusion criteria). We started collecting data in October 2021 and completed data collection in May 2023 after collecting 60 valid datasets for all experiments of the larger project. Since all participants who completed the behavioral or fMRI experiments mentioned above also completed the behavioral tasks of this study, we ended up with more than 60 datasets (we collected 100 datasets in total, 44 patients and 56 controls). We excluded nine patients (one because of task comprehension problems, two because of intake of medication listed in our exclusion criteria, four because they fulfilled less than four IPDE criteria, two because of drug use before testing) and six controls (due to clinically relevant symptoms assessed via the diagnostic interview) before data analysis.

#### Task specific exclusion procedure

We applied additional preregistered exclusion criteria for the different tasks resulting in slightly different sample sizes. For the analysis of the SVO scores and categories, we excluded participants with intransitive choices (see SVO task in Analyses section) in the primary items (25) resulting in a sample of 29 patients and 50 controls. For the analysis of the prosocial motivation scores of the SVO task, we could only include participants with prosocial SVO scores according to both primary and secondary items (see Analyses and 26), resulting in a sample of 24 patients and 44 controls. Although we did not initially specify any exclusion criteria for the UG, we decided to exclude one participant from the control group due to an implausible response pattern which indicates that this participant misunderstood the task (specifically, the participant accepted all unfair offers from 1 to 3 and rejected all fair offers from 4 to 6). Consequently, the sample sizes for the UG analysis were 35 participants in the BPD group and 49 participants in the control group.

We did not have to exclude any of the participants due to implausible response patterns in the Fairness Ratings or the JPE task. In this case, we defined “implausible” as selecting the same choice option more than 90% of the trials in the Fairness Ratings.

#### Ordering and randomization of the tasks

The order of the tasks was not randomized on purpose. We chose a specific order to minimize the influence between the tasks. That is, tasks on social preferences (SVO) and evaluation of allocations (JPE) were presented before participants were asked to actively allocate money between themselves and other participants (DG). Participants performed the active allocation (DG) before responding to allocations from others (UG). We asked them about the minimum offer they would be willing to accept (Minimum Acceptance Rating) after they accepted or rejected several offers (UG). Finally, we asked the participants to rate the fairness of hypothetical offers because we wanted to be able to differentiate between the decisions that participants actually make, and the ones they consider fair.

For the items in the SVO task, we used the two different versions of the task (same items in different order) provided by Murphy (<http://ryanomurphy.com/styled-2/downloads/index.html>). The version of the SVO task given to the participants was based on their individual subject number. The order of the items within the UG task, the JPE task, and the Fairness Ratings were fully randomized. All participants saw the exact same items in a different order. For randomization, we use the MATLAB function *randperm*. All tasks were presented using the MATLAB toolbox Psychtoolbox (psychtoolbox.org). Response time was not restricted in any of the tasks.

### Experimental procedures

Participants were recruited via (online) advertisements, flyers, and existing databases. For the recruitment of participants with BPD, we contacted psychotherapists in Heidelberg and surrounding towns and recruited inpatients at the Department of General Psychiatry at the University of Heidelberg. Healthy participants were paid a minimum of 50€ and participants with BPD were paid a minimum of 60€ (due to differences in the amount of time required for the diagnostic interview) for completing all experiments that are part of the project. In addition, all participants received a bonus depending on their decisions during the experiments. In case participants participated only in some of the experiments, they were paid a standard hourly fee.

#### Tasks

##### SVO task
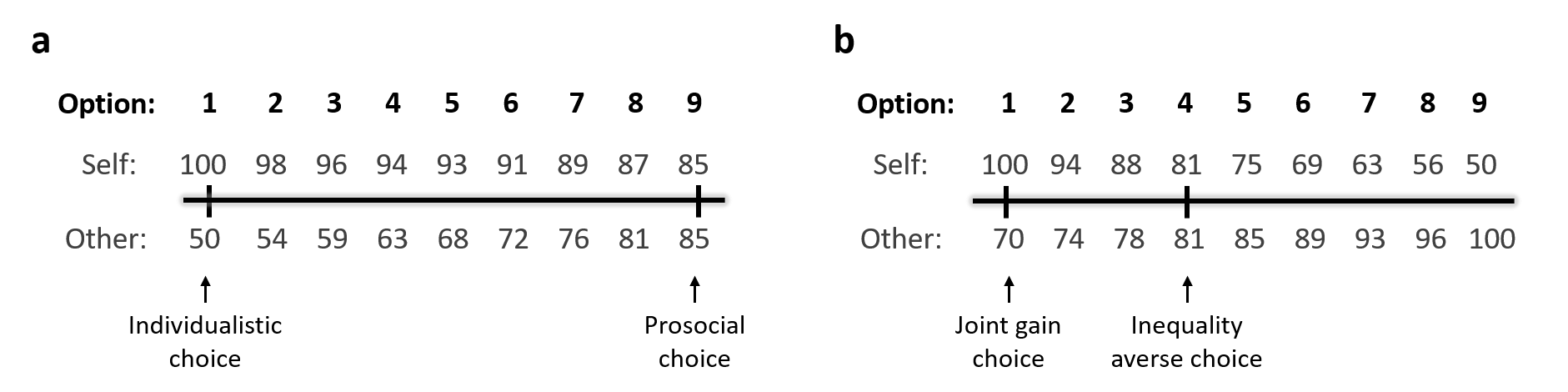


**Supplementary Figure S1.** Example items of the SVO Slider task. (a) Primary item 6 measures general Social Value Orientation and (b) secondary item 9 measures prosocial motivation (two different motives for being prosocial: joint gain maximization and inequality aversion).

## Supplementary Analyses

### Calculation of SVO scores

The items of the SVO task are sampled from an allocation plane defined by two orthogonal dimensions: outcomes for self (participant) and outcomes for other (anonymous participant). The endpoints (options 1 and 9 of each item) lie on a circle centered at 50 with a diameter of 50. The SVO score is an angle on this allocation plane (see Supplementary Material for details of calculation).

Participants with intransitive responses for the primary items were excluded from the analyses. Transitivity implies that if a person prefers choice A over choice B, and choice B over choice C, then they should also prefer choice A over choice C. However, if responses are random, it is highly likely that the choices will be intransitive, meaning they won't follow this logical order of preferences.

To calculate the SVO score, we determined the individual mean allocation for self and the other from the primary items, then subtracted 50 from both means to shift the base of the resulting SVO angle to the center of the circle. The SVO angle for each participant was calculated by taking the inverse tangent of the ratio of the adjusted means. SVO scores can be classified into four categories: competitive (less than -12.04°), individualistic (between -12.04° and 22.45°), prosocial (between 22.45° and 57.15°), and altruistic (more than 57.15°).

For computing the prosocial motivation score from the nine secondary items, we first calculated the “inequality distance score” which quantifies the distance between the participant’s chosen options and the options that would minimize the inequality between the payoffs for self and the other. Second, we calculated the “joint gain distance score,” which quantifies the distance between the participant’s chosen options and those that would maximize joint gain. The prosocial motivation score was derived by dividing the inequality distance score by the sum of the inequality distance score and the joint-gain distance score. This results in a single score ranging from 0 (perfect inequality aversion) to 1 (perfect joint gain maximization). Based on this score, participants can be categorized as either inequality averse (prosocial motivation score < 0.5) or joint gain maximizing (> 0.5). Participants who scored exactly 0.5 are not categorized (26).

### Economic models for the JPE task

We compared the following economic models (see Supplementary Material in 27 for a detailed model description):

In all models, Utility (U) depends on various (combinations of) weighted predictors. In the first five models, utility is determined by the weighted payoff for Self ($\$Self)$ and/or other ($\$Other$), or a combination of these payoffs (e.g., the sum, the joint payoff, and/or the absolute difference between payoffs). The Fehr-Schmidt model (28) separately considers the payoff for Self, Disadvantageous Inequality, and Advantageous Inequality. If the difference between $Other and $Self is negative, the term max(*$Other* - *$Self*,0) sets the result to zero (preserving positive values). The same logic applies to the difference between $Self and $Other (*$Self* - *$Other*). Parameters were not restricted, allowing for the possibility of altruistic and competitive motives. The ERC model (29) accounts for quadratic and normalized inequality, without explicitly distinguishing between advantageous and disadvantageous inequality.

1. Individualism model:
    $U=\alpha*\$Self$
2. Prosocial model:
    $U=\alpha*\$Self+\beta*\$Other$
3. Altruism model:

$$U=\beta*\$Other$$

4. Joint-gain model:
    $U=\alpha*(\$Self+\$Other)$
5. Inequality model:
    $U=\alpha*abs(\$Self-\$Other)$
6. Inequality & Joint-gain model:

$$U=\alpha*abs\left( \$Self-\$Other \right)+ \beta* \left( \$Self+\$Other \right)$$

7. Fehr-Schmidt model:

$$U=\alpha*\$Self+\beta*\max\left( \$Other-\$Self,0 \right)+\gamma*max(\$Self-\$Other,0)$$

8. Fehr-Schmidt model with separate parameters for positive and negative payoffs for self and disadvantageous inequality so that:

$\alpha$ = $\alpha_{pos}$ for $Self $\geq0,$

$\alpha$ = $\alpha_{neg}$ for $Self $<0,$

$\beta$ = $\beta_{pos}$ for $Self $\geq0,$

$\beta$ = $\beta_{neg}$ for $Self $\geq0,$

1. ERC (Equity, Reciprocity, Competition) model:

$$U=\alpha*\$Self+\frac{\beta}{2}*\left( \frac{\$Self}{\$Self+\$Other}-\frac{1}{2} \right)^{2}$$

To assess model evidence, we conducted both fixed-effects analysis (assumption that all participants use the same model) and random-effects analysis (assumption that different participants may use different models). For the fixed-effects analysis, we calculated log-group Bayes factors. We first, calculated the Bayesian information criterion (BIC; 30) for each participant and each model, using the standard formula: BIC = n * ln(RSS/n) + k * ln(n), where RSS is the residual sum of squared errors, n is the number of trials, k is the number of free parameters in the model, and “ln” is the natural logarithm. The BIC penalizes model complexity (i.e., the number of parameters in the model). To compute log-group Bayes factors, we summed the BIC scores across the group and subtracted the BIC of a reference model from the BIC of each tested model (31). The model with the smallest log-group Bayes factor is considered the best fit.

For the random-effects analysis, we calculated protected exceedance probabilities using the Bayesian Model Selection (BMS), a procedure implemented in the MATLAB toolbox SPM12 (http://www.fil.ion.ucl.ac.uk/spm/;spm_BMS). The protected exceedance probability indicates the probability that a given model is more likely across subjects than any other model, accounting for differences in model evidence due to chance (32).

## Supplementary Results

###
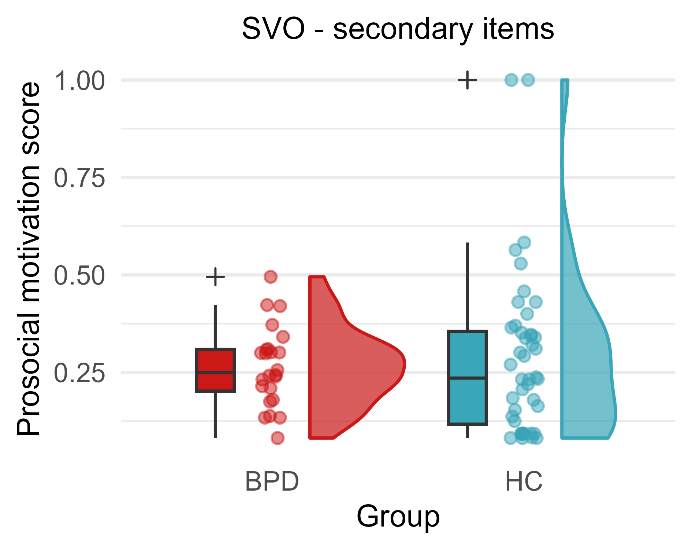
SVO Prosocial Motivation

**Supplementary Figure S2.** Active cooperation task results. Raincloud plots show the distribution of prosocial motivation scores (secondary items of the SVO task) in the BPD group and the HC group.

### Dissociality scores

Since we used dissociality scores to examine their potential association with reduced reactive cooperation in the UG, we also tested whether dissociality scores varied between the groups. We found that participants from the BPD group scored significantly higher (*M_BPD_* = 28.314, *SD_BPD_*= 8.878, *N_BPD_* = 35) than participants form the control group (*M_HC_* = 19.082, *SD_HC_* = 4.559, *N_HC_* = 49; Welch’s two-sample t-test *t_46.839_* = 5.644, *p*< 0.001, 95% *CI* [5.942, 12.524], *d* = 1.31; Wilcoxon rank sum test *z* = 5.157, *p* < 0.001, *r* = 0.563; **Supplementary Figure S3**). Note that the sample size used here is the same as in the UG analysis.

**Supplementary**
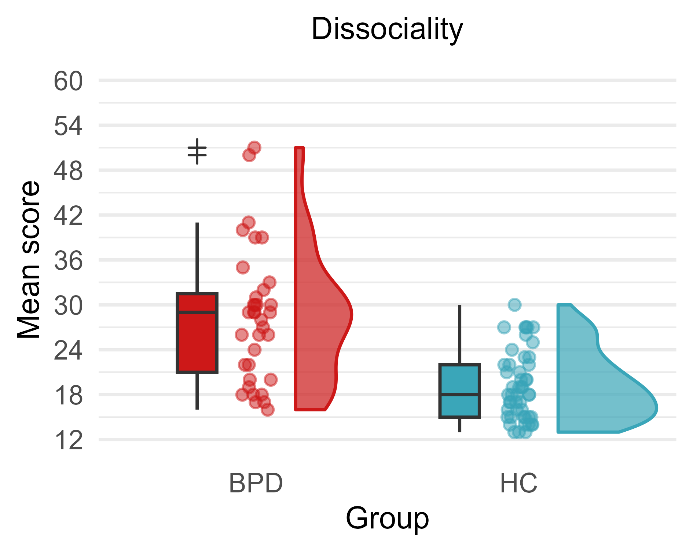
**Figure S3.** Raincloud plots show the distribution of dissociality scores in the BPD group and the HC group. The plot includes individual dissociality scores for each participant, a box plot, and a probability density plot. The boxplot visualizes the median, the 25th and 75th percentiles, two whiskers (extending from the upper and lower hinges to the largest and lowest value within 1.5 times the inter-quartile range, and all outliers individually. The density plot was generated using Gaussian kernel density estimation.

### Fairness Ratings

**Supplementary Table S2.** Means and standard deviations of Fairness Ratings

|  | BPD | | HC | |
| --- | --- | --- | --- | --- |
| Offer range | *M* | *SD* | *M* | *SD* |
| Disadvantageous offers | 1.921 | 0.65 | 2.355 | 1.001 |
| Fair offers | 6.629 | 1.081 | 6.473 | 1.518 |
| Advantageous offers | 3.029 | 1.84 | 2.780 | 1.317 |

### JPE model comparison


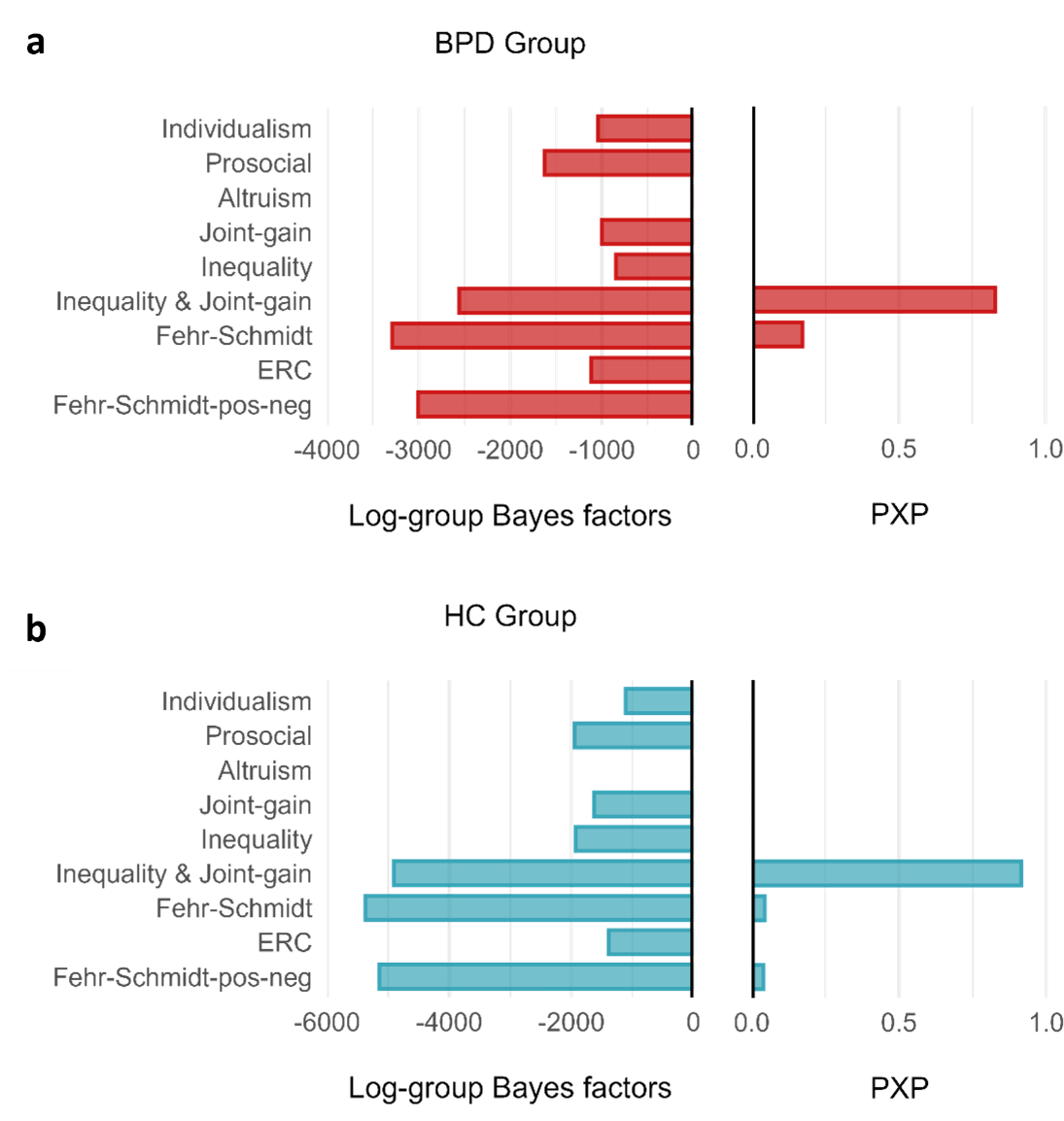
**Supplementary Figure S4.** Model comparisons of the JPE task data according to fixed-effects analyses (Log-group Bayes factors) and random-effects analyses (protected exceedance probabilities, PXPs). Log-group Bayes factors are presented relative to a reference model (Altruism model). Lower numbers indicate better model fit in the Log-group Bayes factors and higher numbers indicate better model fit in the PXPs. The Fehr-Schmidt model performed best in both groups according to the fixed-effects analyses, and the Inequality & Joint-gain model performed best in both groups according to the random-effects analyses.

## Mini Meta-Analysis on Ultimatum Games

### Eligibility criteria

We included studies that met the following criteria: (a) experimental designs, (b) assessment based on a clinical diagnosis of Borderline Personality Disorder (BPD) or BP(D) features, (c) use of a variant of the Ultimatum Game, and (d) design with participants in the responder role. For studies involving diagnosed BPD samples, (e) a comparison with a healthy control group was required. Additionally, (f) the studies had to measure rejections in the Ultimatum Game as the primary outcome. Only studies published in English or German were considered eligible for inclusion. Both published and unpublished studies were included, and we actively searched for gray literature.

### Search strategy and selection

We conducted a comprehensive search for eligible studies using the databases PubMed and PsycINFO (last search on October 3, 2023). The search strings used were “Borderline AND Ultimatum Game”. Due to the limited number of studies found, we extended our search by issuing a call for data through the German Psychological Society (DGPs) and by reaching out to researchers in the same field at conferences. For study selection, we first screened abstracts and subsequently assessed full texts for eligibility based on our predefined criteria. The study selection process is depicted in a flowchart in **Supplementary Figure S5**.

### Data extraction

From each article, we extracted the following data: (a) the first author, (b) the year of publication, (c) the sample size (separated by groups if applicable), (d) BPD feature scores (if applicable), (e) sample characteristics, including whether the study assessed a BPD vs. healthy control (HC) group or a sample with BPD features, along with demographic information such as gender and age, (f) the specific Ultimatum Game outcome measured, (g) data required for effect size calculation, and (h) any additional conditions assessed. If certain information was not directly available in the article, we made up to two attempts to contact the corresponding authors and excluded articles if we didn’t receive a response.

### Meta-Analysis procedure

The meta-analysis was conducted in R (33) using the meta package (34), following the guidelines outlined by Harrer et al. (35). For each study, between-group effect sizes (standardized mean differences) were calculated as Cohen’s d. For the study reporting BPD feature scores, participants were divided into groups based on established cut-off criteria (36). If studies reported outcomes separately for additional conditions, the data were aggregated across these conditions before calculating the effect sizes.

We used a random-effects model because we expected heterogeneity between studies. To calculate the 95% confidence interval for the pooled effect size, we used the Knapp-Hartung adjustments (37), which are recommended for meta-analyses with a small number of studies. Additionally, alongside the heterogeneity statistic I², which can be biased in smaller meta-analyses, we employed the Paule-Mandel estimator (38) to assess heterogeneity variance (τ²). For the targeted outcome—rejections in the Ultimatum Game—all available data were pooled, regardless of whether rejections were the primary or secondary outcome in the studies. To manage potential outliers, we examined if any of the studies´ 95% confidence intervals fell outside the pooled effect size. None of the studies met this criterion, indicating no outliers. Publication bias was evaluated using a funnel plot.

The systematic search initially identified 12 studies. After removing duplicates and excluding records based on abstract screening, five studies were selected for full-text analysis. One study was subsequently excluded due to insufficient data for effect size calculation, as no response was received from the authors. Ultimately, four studies met the inclusion criteria and were included in the final meta-analysis. Detailed characteristics of these studies are presented in **Supplementary Table S3.**


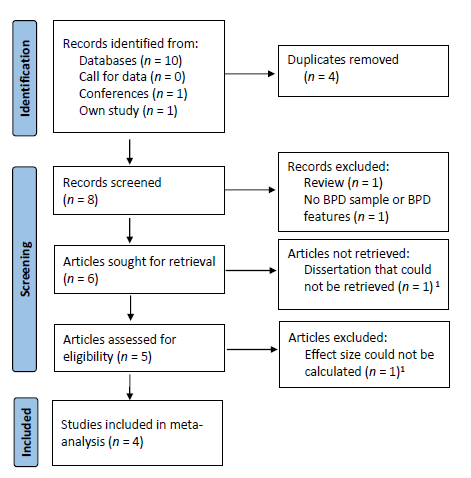


**Supplementary Figure S5.** PRISMA Flowchart of the Study Selection Process.

^1^Authors were contacted and asked for the data needed. For the excluded articles no response was received.

**Supplementary Table S3.** Characteristics of the studies included in the Meta-Analysis

| Study | Sample Type | BPD Measure | *N HC* | *N BPD* | Female (%) | Age (M) | Outcome | Additional Conditions |
| --- | --- | --- | --- | --- | --- | --- | --- | --- |
| De Panfilis et al. (39) | HC, BPD | SCID-II | 41 | 41 | 76.8 | 37 | Percentage of offers rejected^1^ | Emotion regulation strategies |
| Graumann et al. (40) | HC, BPD | SCID | 97 | 94 | 100 | 28 | Percentage of offers rejected^1^ | Overinclusion and exclusion |
| Thielmann et al. (41) | Community sample (BPD features)^2^ | VEI-BOR | 94 | 168 | 82^3^ | 31.2^3^ | Offers rejected after breakdown | Cooperation breakdown severity |
| This Study | HC, BPD | IPDE | 49 | 36 | 100 | 25.8^3^ | Percentage of offers rejected | none |

*Note*. HC = Healthy Control group; BPD = Borderline Personality Disorder group. BPD Group Measure = measure of BPD symptoms or features. SCID/SCID-II = *Structured Clinical Interview* (BPD criterion: 5 out of 9 symptoms), VEI-BOR = *Verhaltens-Erlebens-Inventar* (Cut-off for BPD group inclusion: total score >38), IPDE = *International Personality Disorder Examination* (BPD criterion: 4 out of 9 symptoms). Outcome = outcome that was used for the meta-analysis. Additional conditions = conditions in the original study that varied within or between participants.

^1^Studies reported the outcome separately for additional conditions. For the meta-analysis, data was aggregated across conditions.

^2^No grouped sample, authors recruited participants via online communities, BPD-related web pages, and mailing lists and continuously assessed their BPD features.

^3^Studies reported descriptives for the whole sample, but only a sub-sample was included in the task for the target outcome. This was because of separate exclusion criteria or because only a sub-sample got assigned the condition with the target outcome in designs with different tasks.


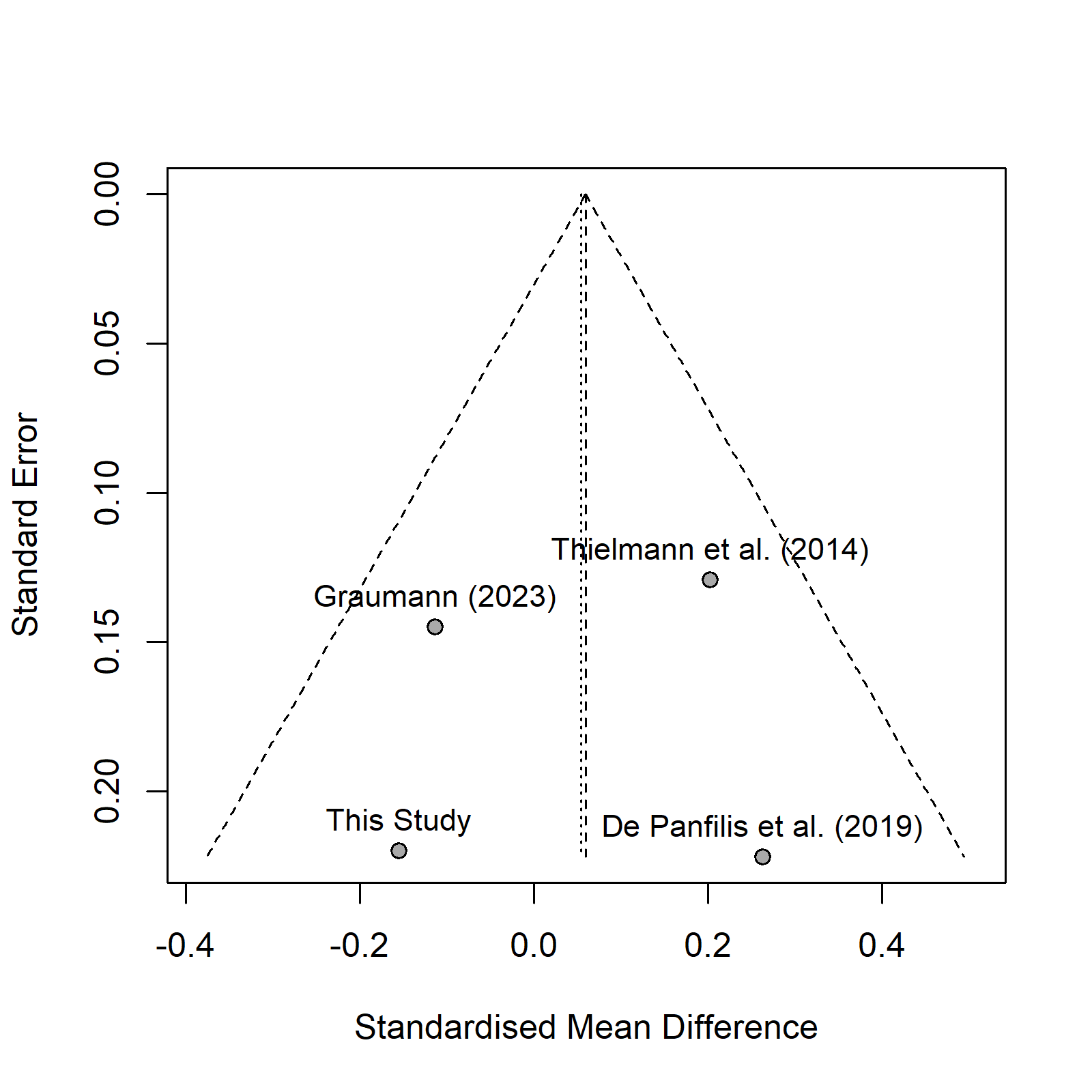
**Supplementary Figure S6**. Funnel Plot for Meta-Analysis. The effect size of each study is depicted on the x-axis and the respective standard error on the y-axis. The vertical line represents the average effect size. All studies are within the funnel. Studies with a lower standard error lie closer to each other, resulting in an overall symmetrical pattern not indicative of publication bias.

## Supplementary References

1. Margraf J, Cwik JC. Mini-DIPS Open Access: Diagnostisches Kurzinterview bei psychischen Störungen. 2017 [cited 2022 May 17]; Available from: https://omp.ub.rub.de/index.php/RUB/catalog/book/102

2. Loranger AW. The International Personality Disorder Examination: The World Health Organization/Alcohol, Drug Abuse, and Mental Health Administration International Pilot Study of Personality Disorders. Arch Gen Psychiatry. 1994 Mar 1;51(3):215.

3. Bohus M, Kleindienst N, Limberger MF, Stieglitz RD, Domsalla M, Chapman AL, et al. The Short Version of the Borderline Symptom List (BSL-23): Development and Initial Data on Psychometric Properties. Psychopathology. 2009;42(1):32–9.

4. Franke GH. BSCL-53®-S. Brief symptom-checklist–standard–German manual. Göttingen: Hogrefe Publishing Group; 2017.

5. Beck AT, Steer RA, Brown GK. BDI-II. Beck-Depressionsinventar [Beck Depression Inventory II]. Frankfurt (Germany): Pearson Verlag; 2006.

6. Kessler RC, Adler L, Ames M, Demler O, Faraone S, Hiripi E, et al. The World Health Organization adult ADHD self-report scale (ASRS): a short screening scale for use in the general population. Psychol Med. 2005 Feb;35(2):245–56.

7. Rösler M, Retz W, Retz-Junginger P, Thome J, Supprian T, Nissen T, et al. Instrumente zur Diagnostik der Aufmerksamkeitsdefizit-/Hyperaktivitätsstörung (ADHS) im Erwachsenenalter: Selbstbeurteilungsskala (ADHS-SB) und Diagnosecheckliste (ADHS-DC). Nervenarzt [Internet]. 2004 Oct [cited 2022 May 17];75(9). Available from: http://link.springer.com/10.1007/s00115-003-1622-2

8. Retz-Junginger P, Retz W, Blocher D, Weijers HG, Trott GE, Wender PH, et al. Wender Utah Rating Scale (WURS-k) Die deutsche Kurzform zur retrospektiven Erfassung des hyperkinetischen Syndroms bei Erwachsenen: Die deutsche Kurzform zur retrospektiven Erfassung des hyperkinetischen Syndroms bei Erwachsenen. Nervenarzt. 2002 Sep;73(9):830–8.

9. Steyer R, Schwenkmezger P, Notz P, Eid M. The multidimensional mental state questionnaire: Manual. Göttingen: Hogrefe; 1997.

10. Borkenau P, Ostendorf F. NEO-Fünf-Faktoren-Inventar nach Costa und McCrae (NEO-FFI; 2. neu normierte und vollständig überarb. Aufl.). Göttingen: Hogrefe; 2007.

11. Bach B, Kerber A, Aluja A, Bastiaens T, Keeley JW, Claes L, et al. International Assessment of DSM-5 and ICD-11 Personality Disorder Traits: Toward a Common Nosology in DSM-5.1. Psychopathology. 2020;53(3–4):179–88.

12. Zimmermann J, Altenstein D, Krieger T, Holtforth MG, Pretsch J, Alexopoulos J, et al. The Structure and Correlates of Self-Reported DSM-5 Maladaptive Personality Traits: Findings From Two German-Speaking Samples. J Personal Disord. 2014 Aug;28(4):518–40.

13. Rohrmann S, Hodapp V, Schnell K, Tibubos AN, Schwenkmezger P, Spielberger CD. Das State-Trait-Ärgerausdrucks-Inventar-2 (STAXI-2). Deutschsprachige Adaptation des State-Trait Anger Expression Inventory-2 (STAXI-2) von Charles D. Spielberger [State-Trait Anger-Expression-Inventory-2 (STAXI-2). German Adaptation of the State-Trait-Anger-Expression-Inventory-2 (STAXI-2) of Charles D. Spielberger]. Bern: Hans Huber; 2013.

14. Preuss UW, Rujescu D, Giegling I, Watzke S, Koller G, Zetzsche T, et al. Psychometrische Evaluation der deutschsprachigen Version der Barratt-Impulsiveness-Skala. Nervenarzt. 2008 Mar;79(3):305–19.

15. Hofmann SG, Kashdan TB. The Affective Style Questionnaire: Development and Psychometric Properties. J Psychopathol Behav Assess. 2010 Jun;32(2):255–63.

16. Pruessner L, Holt DV, Gölz R, Sevcenko N, Hofmann SG, Backenstrass M. Psychometrische Eigenschaften der deutschsprachigen Version des Interpersonal Emotion Regulation Questionnaire. Diagnostica. 2020 Jan;66(1):62–73.

17. Baudson TG, Preckel F. mini-q: Intelligenzscreening in drei Minuten. Diagnostica. 2016 Jul;62(3):182–97.

18. Damovsky F, Zettl M, Zimmermann J, Herbold W, Curtius T, Buecker S, et al. Das Persönlichkeitsinventar für ICD-11: Reliabilität und Validität der deutschen Version in einer klinischen und nicht-klinischen Stichprobe [Internet]. Open Science Framework; 2021 Sep [cited 2022 May 17]. Available from: https://osf.io/cm3qv

19. Oltmanns JR, Widiger TA. A self-report measure for the ICD-11 dimensional trait model proposal: The Personality Inventory for ICD-11. Psychol Assess. 2018 Feb;30(2):154–69.

20. Spitzer C, Müller S, Kerber A, Hutsebaut J, Brähler E, Zimmermann J. Die deutsche Version der Level of Personality Functioning Scale-Brief Form 2.0 (LPFS-BF): Faktorenstruktur, konvergente Validität und Normwerte in der Allgemeinbevölkerung. PPmP - Psychother · Psychosom · Med Psychol. 2021 Jul;71(07):284–93.

21. Weekers LC, Hutsebaut J, Kamphuis JH. The Level of Personality Functioning Scale-Brief Form 2.0: Update of a brief instrument for assessing level of personality functioning: The Level of Personality Functioning Scale - Brief Form 2.0. Personal Ment Health. 2019 Feb;13(1):3–14.

22. Kröger C, Kosfelder J. IES-27: Skala zur Erfassung der Impulsivität und emotionalen Dysregulation der Borderline-Persönlichkeitsstörung. Göttingen: Hogrefe; 2011.

23. Faul F, Erdfelder E, Lang AG, Buchner A. G*Power 3: A flexible statistical power analysis program for the social, behavioral, and biomedical sciences. Behav Res Methods. 2007 May;39(2):175–91.

24. Korn CW, La Rosée L, Heekeren HR, Roepke S. Social feedback processing in borderline personality disorder. Psychol Med. 2016 Feb;46(3):575–87.

25. Leiner DJ. SoSci Survey (Version 3.1.06) [Internet]. 2019. Available from: https://www.soscisurvey.de

26. Murphy RO, Ackermann KA, Handgraaf M. Measuring Social Value Orientation. SSRN Electron J [Internet]. 2011 [cited 2019 Jul 23]; Available from: http://www.ssrn.com/abstract=1804189

27. Doppelhofer LM, Hurlemann R, Bach DR, Korn CW. Social motives in a patient with bilateral selective amygdala lesions: Shift in prosocial motivation but not in social value orientation. Neuropsychologia. 2021 Nov;162:108016.

28. Fehr E, Schmidt KM. A Theory of Fairness, Competition, and Cooperation. Q J Econ. 1999 Aug 1;114(3):817–68.

29. Bolton GE, Ockenfels A. ERC: A Theory of Equity, Reciprocity, and Competition. Am Econ Rev. 2000 Mar;90(1):166–93.

30. Schwarz G. Estimating the Dimension of a Model. Ann Stat. 1978;6(2):461–4.

31. Stephan KE, Penny WD, Daunizeau J, Moran RJ, Friston KJ. Bayesian model selection for group studies. NeuroImage. 2009 Jul;46(4):1004–17.

32. Rigoux L, Stephan KE, Friston KJ, Daunizeau J. Bayesian model selection for group studies — Revisited. NeuroImage. 2014 Jan;84:971–85.

33. R Core Team. R: A Language and Environment for Statistical Computing [Internet]. Vienna, Austria: R Foundation for Statistical Computing; 2024. Available from: https://www.R-project.org/

34. Schwarzer G, Carpenter JR, Rücker G. Meta-Analysis with R [Internet]. Cham: Springer International Publishing; 2015 [cited 2024 Oct 8]. (Use R!). Available from: https://link.springer.com/10.1007/978-3-319-21416-0

35. Harrer M, Cuijpers P, Furukawa TA, Ebert DD. Doing Meta-Analysis with R: A Hands-On Guide [Internet]. 1st ed. Boca Raton: Chapman and Hall/CRC; 2021 [cited 2024 Oct 8]. Available from: https://www.taylorfrancis.com/books/9781003107347

36. Hepp J, Hilbig BE, Kieslich PJ, Herzog J, Lis S, Schmahl C, et al. Borderline Personality and the Detection of Angry Faces. Felmingham K, editor. PLOS ONE. 2016 Mar 31;11(3):e0152947.

37. Knapp G, Hartung J. Improved tests for a random effects meta‐regression with a single covariate. Stat Med. 2003 Sep 15;22(17):2693–710.

38. Paule RC, Mandel J. Consensus Values and Weighting Factors. J Res Natl Bur Stand. 1982 Sep;87(5):377.

39. De Panfilis C, Schito G, Generali I, Gozzi LA, Ossola P, Marchesi C, et al. Emotions at the border: Increased punishment behavior during fair interpersonal exchanges in borderline personality disorder. J Abnorm Psychol. 2019 Feb;128(2):162–72.

40. Graumann L, Kulakova E, Cho AB, Deuter CE, Wolf OT, Schell J, et al. Elevated testosterone and prosocial behavior in female patients with borderline personality disorder independent of social exclusion. Psychoneuroendocrinology. 2025 Jan;171:107232.

41. Thielmann I, Hilbig BE, Niedtfeld I. Willing to Give but Not to Forgive: Borderline Personality Features and Cooperative Behavior. J Personal Disord. 2014 Dec;28(6):778–95.
